# The multi-level and multi-dimensional quantum wavelet packet transforms

**DOI:** 10.1038/s41598-018-32348-8

**Published:** 2018-09-17

**Authors:** Hai-Sheng Li, Ping Fan, Hai-ying Xia, Shuxiang Song, Xiangjian He

**Affiliations:** 1 Guangxi Normal University, College of Electronic Engineering, Guilin, 541004 China; 2 East China JiaoTong University, College of Information Engineering, Nanchang, 330013 China; 3 University of Technology, Sydney, School of Electrical and Data Engineering, Sydney, NSW 2000 Australia

## Abstract

The classical wavelet packet transform has been widely applied in the information processing field. It implies that the quantum wavelet packet transform (QWPT) can play an important role in quantum information processing. In this paper, we design quantum circuits of a generalized tensor product (GTP) and a perfect shuffle permutation (PSP). Next, we propose multi-level and multi-dimensional (1D, 2D and 3D) QWPTs, including a Haar QWPT (HQWPT), a D4 QWPT (DQWPT) based on the periodization extension and their inverse transforms for the first time, and prove the correctness based on the GTP and PSP. Furthermore, we analyze the quantum costs and the time complexities of our proposed QWPTs and obtain precise results. The time complexities of HQWPTs is at most 6 on 2^*n*^ elements, which illustrates high-efficiency of the proposed QWPTs. Simulation experiments demonstrate that the proposed QWPTs are correct and effective.

## Introduction

With the rapid development in the fields of optical imaging, Internet technology, high performance calculation etc., the amount of data is increasing explosively, so that it is necessary to find new ways to store and process information. Quantum information processing (QIP)^1^ as new technology of information processing, offers a potential solution to store and process massive visual data efficiently. QIP has two outstanding merits: (1) the unique computing performance of quantum coherence, entanglement and superposition [1], and (2) quantum storage capacity increasing exponentially. Models of quantum image representation^2–8^ have displayed the enormous storage capacity of QIP. Other popular quantum algorithms, such as the Shor’s discrete logarithms and integer-factoring algorithms^9^, the Deutsch’s parallel computing algorithm^10^ and the Grover’s quadratic speed up algorithm^11^, have further shown that QIP is more efficient than its classical counterparts. In addition, many algorithms of QIP emerge continually, and these algorithms include quantum geometric transformation^12–14^, quantum image encryption and decryption algorithms^15,16^, quantum watermarking^17^, quantum image compression^6^, quantum edge detection^18^, and quantum image filtering^19^.

The classical wavelet packet transform (WPT) has been widely spread to the information processing field for image coding^20^, pattern matching^21^ and fractional brownian motion decorrelation^22^. It indicates that the quantum wavelet packet transform (QWPT) plays an important role in QIP. Unfortunately, the research on QWPT is rare and still preliminary. For example, two important QWPTs, namely the Haar QWPT (HQWPT) and the D4 QWPT (DQWPT) proposed in^23–26^, are still single level quantum wavelet transforms. Up to now, we have not yet found any implementation of a multi-level and multi-dimensional QWPT. Therefore, we believe that QWPTs deserve further research.

In this paper, we introduce the generalized tensor product (GTP) and the perfect shuffle permutation (PSP), and design quantum circuits for them. Then, we propose the iterations and implementation circuits of the multi-level and multi-dimensional QWPT and inverse QWPT (IQWPT). QWPTs and the inverse QWPTs being considered include HQWPT, DQWPT based on a periodization extension, the inverse HQWPT (IHQWPT), the inverse DQWPT (IDQWPT). In addition, we analyze the quantum costs and time complexities of the proposed circuits and prove that the multi-level and multi-dimensional HQWPT can be implemented with a complexity of *O*(1). Simulation experiments demonstrate that the proposed QWPTs are correct and effective.

The contributions of this paper are listed as follows.

- We analyze precisely the complexities of the simulated networks of controlled NOT gates with multi-control qubits. Comparing with the methods proposed in the reference^27^, our proposed simulated networks are reduced by 50% approximately.
- We design the simplified circuits of the PSP and reduce time complexity to 6 for 2^*n*^ elements.
- We present the multi-level and multi-dimensional QWPTs, including HQWPT, IHQWPT, DQWPT and IDQPT for the first time, and prove the correctness by theoretical derivations and simulation experiments.
- We design the circuits of the multi-level and multi-dimensional HQWPT with the complexity *O*(1), which has the overwhelming advantage over the classic Haar WPT.

## The Quantum Implementation of GTP

Let *A* be an *n* × *n* matrix and *B* be an *m* × *m* matrix, then the tensor product $A\otimes B$ is an *mn* × *mn* block matrix in the following equation,

1 $$A\otimes B=[\begin{array}{ccc}{A}_{0,0}B & \cdots  & {A}_{0,n-1}B\\ \vdots  & \ddots  & \vdots \\ {A}_{n-1,0}B & \cdots  & {A}_{n-1,n-1}B\end{array}].$$

Thus, the tensor product of quantum states are defined as the tensor product of matrices: $|u\rangle \otimes |v\rangle ={[{u}_{0}\cdots {u}_{{2}^{n}-1}]}^{T}\otimes {[{v}_{0}\cdots {v}_{{2}^{n}-1}]}^{T}$, which is also written simply as $|u\rangle$ $|v\rangle$ or $|uv\rangle$.

Then, *n* fold tensor product $U\otimes U\otimes \cdots \otimes U$ is abbreviated as ${U}^{\otimes n}$. Similarly, the abbreviation of $|u\rangle \otimes |u\rangle \otimes \cdots \otimes |u\rangle$ is ${|u\rangle }^{\otimes n}$.

A larger vector space can be formed by putting vector spaces together. For instance, suppose that $|i\rangle$ is a basic state in a 2^*n*^ dimensional Hilbert space for *i* = 0, 1, …, 2^*n*^ − 1, the state $|i\rangle$ consists of the tensor products of the *n* computation basis states:

2 $$|i\rangle =|{i}_{n}\rangle \otimes |{i}_{n-1}\rangle \otimes \cdots \otimes |{i}_{1}\rangle =|{i}_{n}\rangle |{i}_{n-1}\rangle \cdots |{i}_{1}\rangle =|{i}_{n}{i}_{n-1}\ldots {i}_{1}\rangle ,$$

where $i={\sum }_{j=1}^{n}\,{i}_{j}\times {2}^{j-1}$ and *i*_1_, *i*_2_, …, *i*_*n*_ ∈ {0, 1}. Its dual state is

3 $$\langle i|=\langle {i}_{n}|\otimes \langle {i}_{n-1}|\otimes \cdots \otimes \langle {i}_{1}|=\langle {i}_{n}|\langle {i}_{n-1}|\cdots \langle {i}_{1}|=\langle {i}_{n}{i}_{n-1}\ldots {i}_{1}|.$$

**Figure 1:**
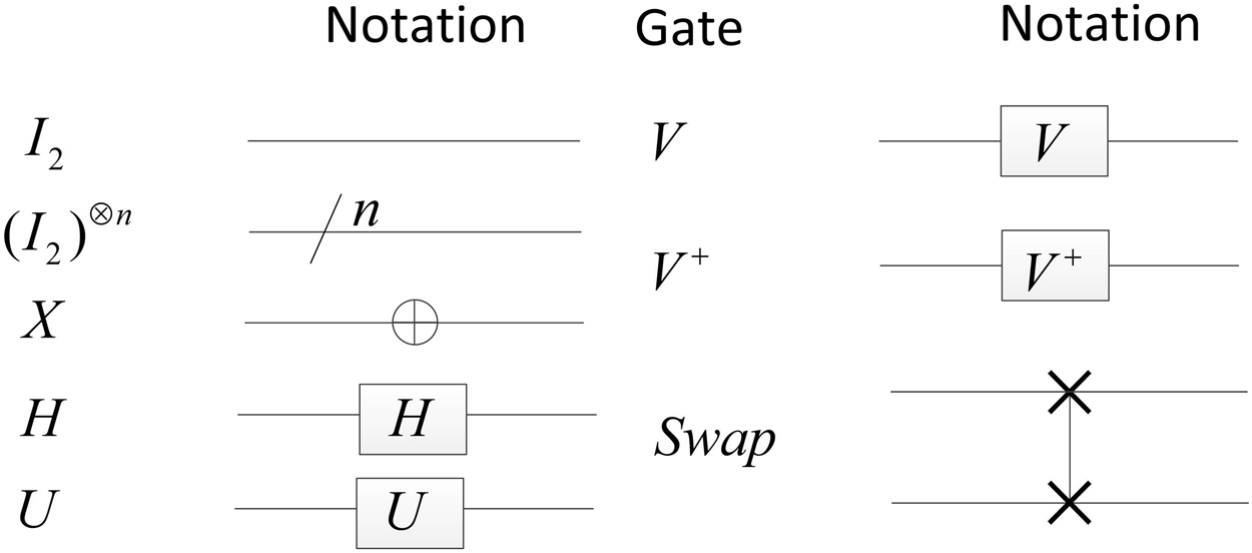
Notations for some base gates with their corresponding symbols.

There are some base gates and their corresponding symbols shown in Fig. 1. In the figure, the identity (*I*_2_), Hadamard (*H*), Pauli-X (*X*) and Swap gates are well-known and can be found in the reference^28^. The 2^*n*^ × 2^*n*^ identity matrix ${({I}_{2})}^{\otimes n}={I}_{{2}^{n}}$ denotes the circuit of *n* qubits. *V* and *V*^+^ are two specific examples of *U* gates where *U* corresponds to a unitary matrix and

4 $$V=\frac{1+i}{2}[\begin{array}{cc}1 & -i\\ -i & 1\end{array}],\,{V}^{+}=\frac{1-i}{2}[\begin{array}{cc}1 & i\\ i & 1\end{array}]\mathrm{.}$$

A controlled gate is one of the most useful gates in quantum computing, and we define two controlled gates of (*n* + *m*)-qubits.

### **Definition 1**.

*Let* ${U}_{{2}^{m}}$ *be a* 2^*m*^ × 2^*m*^ *unitary matrix*, ${I}_{{2}^{m}}$ *be a* 2^*m*^ × 2^*m*^ *identity matrix*. *Then*, *controlled gates* ${C}_{n}^{j}({U}_{{2}^{m}})$ *and* ${V}_{n}^{j}({U}_{{2}^{m}})$ *with n control qubits and m target qubits are defined by*

5 $${C}_{n}^{j}({U}_{{2}^{m}})=(|j\rangle \langle j|)\otimes {U}_{{2}^{m}}+\sum _{i=0,i\ne j}^{{2}^{n}-1}\,((|i\rangle \langle i|)\otimes {I}_{{2}^{m}}),$$

6 $${V}_{n}^{j}({U}_{{2}^{m}})=({U}_{{2}^{m}}\otimes |\,j\rangle \langle \,j|)+\sum _{i=0,i\ne j}^{{2}^{n}-1}\,({I}_{{2}^{m}}\otimes (|i\rangle \langle i|)),$$

**Figure 2:**
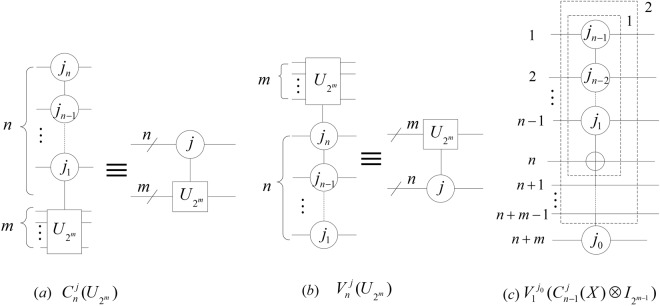
The (*n* + *m*) qubit controlled gates and the ${N}_{n+m}^{n}$ gate. The abbreviation notations are in the right parts of (**a**,**b**). The dashed box 1 and 2 in (**c**) implement ${C}_{n-1}^{j}(X)$ and ${C}_{n-1}^{j}(X)\otimes {I}_{{2}^{m-1}}$ where *j* = *j*_*n*−1_ *j*_*n*−2_ … *j*_1_ and *j*_*n*−1_, *j*_*n*−2_, … *j*_1_, *j*_0_ ∈ {0, 1}.

*where* $|i\rangle =|{i}_{n}\,\cdots \,{i}_{2}{i}_{1}\rangle$ *and* $|j\rangle =|{j}_{n}\,\cdots \,{j}_{2}{j}_{1}\rangle$ *are the basic states in a* 2^*n*^ *dimensional Hilbert space shown in* Eq. (2), *and j* ∈ {0, 1, …, 2^*n*^ − 1}. *The Notations of* ${C}_{n}^{j}({U}_{{2}^{m}})$ *and* ${V}_{n}^{j}({U}_{{2}^{m}})$ *are shown in* (*a*) *and* (*b*) *of* Fig. 2. *Furthermore*, ${C}_{2}^{j}(X)$ *and* ${V}_{2}^{j}(X)$ *are called Toffoli gates*.

### **Definition 2**.

**Figure 3:**
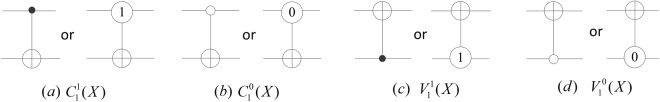
The four ${N}_{2}^{1}$ gates. The numbers 1 and 0 can be replaced by black and white points on control qubits.

*An* (*n* + *m*) *qubit controlled gate with n control qubits is named as an* ${N}_{n+m}^{n}$ *gate*, *when the X gate is in the target qubit of the controlled gate*. *An instance of an* ${N}_{n+m}^{n}$ *gate is shown in* (*c*) *of* Fig. 2. *In addition*, *the four* ${N}_{2}^{1}$ *gates shown in* Fig. 3 *are called controlled*-*NOT gates*.

A Swap gate can be simulated by three ${N}_{2}^{1}$ gates, that is, $Swap={C}_{1}^{0}(X){V}_{1}^{0}(X){C}_{1}^{0}(X)$.

Next, we introduce a perfect shuffle permutation. Let *P*_*n*,*m*_ be the *mn* × *mn* matrix of a perfect shuffle permutation, then *P*_*n*,*m*_ satisfies that (*P*_*n*,*m*_)_*k*,*l*_ = *δ*_*v*,*z*′_*δ*_*z*,*v*′_ where *k* = *vn* + *z*, *l* = *v*′*m* + *z*′, 0 ≤ *v*, *z*′ < *m*, 0 ≤ *v*′, *z* < *n*, *δ*_*x*,*y*_ is the Kronecker delta function, that is, *δ*_*x*,*y*_ = 0 if *x* ≠ *y*, otherwise *δ*_*x*,*y*_ = 1. Therefore, *P*_*n*,*m*_ shuffles *n* packs of *m* cards into *m* packs of *n* cards.

As a useful tool for wavelet transforms, the GTP is defined as follows^29^. Suppose that ${\mathscr{A}}=\{{A}^{0},{A}^{1},\ldots ,{A}^{m-1}\}$ and ${\mathcal B} =\{{B}^{0},{B}^{1},\ldots ,{B}^{n-1}\}$ are two sets of matrices, where *A*^*i*^ is an *n* × *n* matrix, 0 ≤ *i* < *m*, and *B*^*j*^ is an *m* × *m* matrix, 0 ≤ *j* < *n*. Then, the generalized tensor product $C={\mathscr{A}}\otimes  {\mathcal B}$ is an *mn* × *mn* matrix and can be calculated by

7 $$C={\mathscr{A}}\otimes  {\mathcal B} ={P}_{m,n}Diag({\mathscr{A}}){P}_{n,m}Diag( {\mathcal B} ),$$

where $Diag({\mathscr{A}})=Diag({A}^{0},{A}^{1},\ldots ,{A}^{m-1})$ and $Diag( {\mathcal B} )=Diag({B}^{0},{B}^{1},\ldots ,{B}^{n-1})$ are block diagonal matrices.

### **Definition 3**.

*Let* ${\mathscr{A}}=\{{A}^{0},{A}^{1},\ldots ,{A}^{m-1}\}$ *and* ${\mathscr{D}}=\{{D}^{0},{D}^{1},\ldots ,{D}^{m-1}\}$ *be two sets of matrices where A*^*i*^ *and D*^*i*^ *are n* × *n matrices*. *Then*, *the generalized product is defined as* ${\mathscr{A}}\times {\mathscr{D}}={\mathscr{A}}{\mathscr{D}}=\{{A}^{0}\times {D}^{0},{A}^{1}\times {D}^{1},\ldots ,{A}^{m-1}\times {D}^{m-1}\}$.

### **Definition 4**.

*The transpose*, *conjugate transpose and inverse of the matrix set* ${\mathscr{A}}$ *are defined as follows*:

$$\{\begin{array}{l}{{\mathscr{A}}}^{T}=\{{({A}^{0})}^{T},{({A}^{1})}^{T},\ldots ,{({A}^{m-1})}^{T}\},\\ {{\mathscr{A}}}^{+}=\{{({A}^{0})}^{+},{({A}^{1})}^{+},\ldots ,{({A}^{m-1})}^{+}\},\\ {{\mathscr{A}}}^{-1}=\{{({A}^{0})}^{-1},{({A}^{1})}^{-1},\ldots ,{({A}^{m-1})}^{-1}\},\end{array}$$

*where* (*A*^*i*^)^*T*^, (*A*^*i*^)^+^ *and* (*A*^*i*^)^−1^ *denote the transpose*, *conjugate transpose and inverse of matrix A*^*i*^, *respectively*.

The following equations hold by using equation (7) and definitions 3 and 4.

8 $$\{\begin{array}{l}{({\mathscr{A}}\otimes  {\mathcal B} )}^{T}={P}_{m,n}({ {\mathcal B} }^{T}\otimes {{\mathscr{A}}}^{T}){P}_{n,m},\\ {({\mathscr{A}}\otimes  {\mathcal B} )}^{+}={P}_{m,n}({ {\mathcal B} }^{+}\otimes {{\mathscr{A}}}^{+}){P}_{n,m},\\ {({\mathscr{A}}\otimes  {\mathcal B} )}^{-1}={P}_{m,n}({ {\mathcal B} }^{-1}\otimes {{\mathscr{A}}}^{-1}){P}_{n,m}.\end{array}$$

Let ${\mathscr{A}}$ and ${\mathscr{C}}$ be two sets of matrices containing *m* matrices with size *n* × *n*, ${\mathcal B}$ and ${\mathscr{D}}$ be two sets of matrices containing *n* matrices with size *m* × *m*, and *I*_*m*_ and *I*_*n*_ be *m* × *m* and *n* × *n* identity matrices, respectively. Then, the following equation holds^24^:

9 $$({\mathscr{A}}\times {\mathscr{C}})\otimes ( {\mathcal B} \times {\mathscr{D}})=({\mathscr{A}}\otimes {I}_{m})\times ({\mathscr{C}}\otimes  {\mathcal B} )\times ({I}_{n}\otimes {\mathscr{D}}),$$

and implies

10 $$\{\begin{array}{l}{\mathscr{A}}\otimes {\mathscr{D}}=({\mathscr{A}}\times {I}_{n})\otimes ({I}_{m}\times {\mathscr{D}})=({\mathscr{A}}\otimes {I}_{m})\times ({I}_{n}\otimes {\mathscr{D}}),\\ ({\mathscr{A}}\times {\mathscr{C}})\otimes {I}_{m}=({\mathscr{A}}\otimes {I}_{m})\times ({\mathscr{C}}\otimes {I}_{m}).\end{array}$$

Furthermore, calculating by the definition of a GTP, we can implement the following four GTPs using controlled gates:

11 $$\{\begin{array}{l}{I}_{2}\otimes \{{I}_{{2}^{n}},{U}_{{2}^{n}}\}=(|0\rangle \langle 0|)\otimes {I}_{{2}^{n}}+(|1\rangle \langle 1|)\otimes {U}_{{2}^{n}}={C}_{1}^{1}({U}_{{2}^{n}}),\\ {I}_{2}\otimes \{{U}_{{2}^{n}},{I}_{{2}^{n}}\}=(|0\rangle \langle 0|)\otimes {U}_{{2}^{n}}+(|1\rangle \langle 1|)\otimes {I}_{{2}^{n}}={C}_{1}^{0}({U}_{{2}^{n}})\\ \{{I}_{{2}^{n}},{U}_{{2}^{n}}\}\otimes {I}_{2}={I}_{{2}^{n}}\otimes (|0\rangle \langle 0|)+{U}_{{2}^{n}}\otimes (|1\rangle \langle 1|)={V}_{1}^{1}({U}_{{2}^{n}}),\\ \{{U}_{{2}^{n}},{I}_{{2}^{n}}\}\otimes {I}_{2}={U}_{{2}^{n}}\otimes (|0\rangle \langle 0|)+{I}_{{2}^{n}}\otimes (|1\rangle \langle 1|)={V}_{1}^{0}({U}_{{2}^{n}}).\end{array}$$

## The Complexity Analysis of Quantum Circuits

### The complexity analysis of quantsssum circuits

Since a quantum circuit can be simulated by basic operations referring to single-qubit gates, controlled-NOT gates, controlled-*V* and controlled-*V*^+^ gates^12,27,28,30^, we introduce some definitions and lemmas. Furthermore, $\lfloor \rfloor$ and $\lceil \rceil$ are the symbols of round down and round up respectively, which are used in the following definitions and lemmas.

#### **Definition 5**.

*The quantum cost of a quantum circuit can be regarded as the total number of basic operations which simulate the circuit*, *marked by* ${\rm{C}}()$.

#### **Definition 6**.

*The time complexity of a quantum circuit is defined by the total number of time steps*. *In a time step*, *only one basic operation is executed serially*, *but multiple ones can be performed in parallel*. *It is marked by* ${{\rm{C}}}^{p}()$.

#### **Lemma 1**.

*When n* ≥ 6 *and* $m\in \{3,4,\ldots ,\lfloor n/2\rfloor \}$, *an* ${N}_{n}^{m}$ *gate can be simulated by a network consisting of* 2(*m* − 1) *Toffoli gates and a basic operation*.

**Figure 4:**
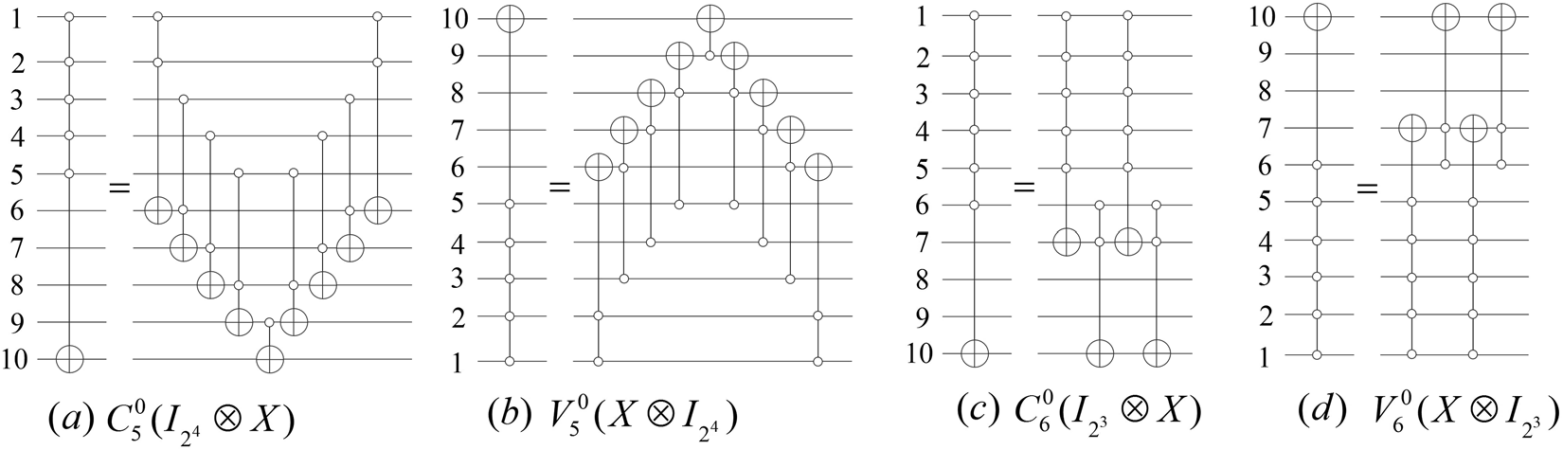
The controlled gate ${N}_{n}^{m}$ illustrated for *n* = 10 and *m* ∈ {5, 6}.

For instance, ${C}_{m}^{0}({I}_{{2}^{n-m-1}}\otimes X)$ and ${V}_{m}^{0}(X\otimes {I}_{{2}^{n-m-1}})$ gates can be simulated by 2(*m* − 1) Toffoli gates and a basic operation, respectively. The form of the network is shown in (a) and (b) of Fig. 4.

#### **Lemma 2**.

*For any n* ≥ 6, $r=\lfloor n/2\rfloor$ *and m* ∈ {*r* + 1, *r* + 2, …, *n* − 2}, *an* ${N}_{n}^{m}$ *gare can be simulated by two* ${N}_{n}^{r}$ *gates and two* ${N}_{n}^{m-r+1}$ *gates*.

For instance, the simulated networks of ${C}_{6}^{0}({I}_{{2}^{3}}\otimes X)$ and ${V}_{6}^{0}(X\otimes {I}_{{2}^{3}})$ gates are shown in (c) and (d) of Fig. 4.

#### **Lemma 3**.

*When n* ≥ 5 *and* $m=\lceil n/2\rceil$, *an* ${N}_{n}^{m}$ *gate can be simulated by a network consisting of* 4(*m* − 2) *Toffoli gates*.

**Figure 5:**
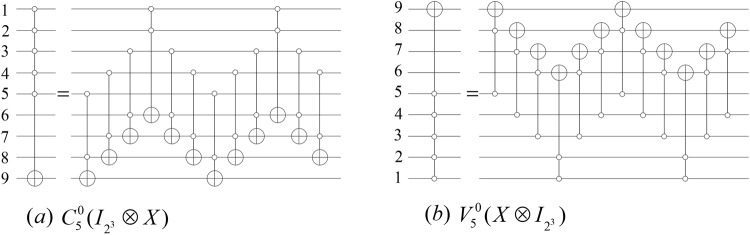
The controlled gate ${N}_{n}^{m}$ illustrated for *n* = 9 and *m* = 5.

For instance, the simulated networks of ${C}_{m}^{0}({I}_{{2}^{n-m-1}}\otimes X)$ and ${V}_{m}^{0}(X\otimes {I}_{{2}^{n-m-1}})$ gates are shown in Fig. 5.

#### **Lemma 4**.

**Figure 6:**
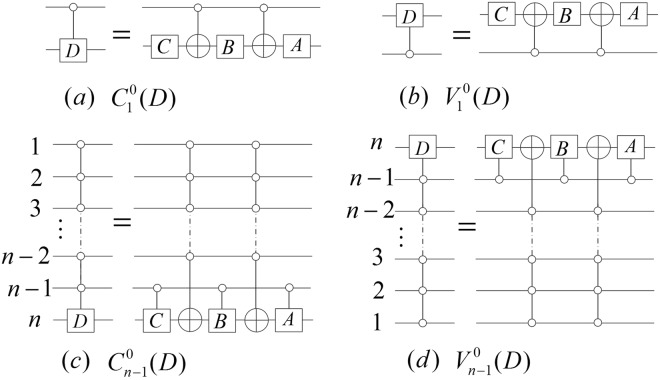
The simulated networks of the gates ${C}_{n-1}^{0}(D)$ and ${V}_{n-1}^{0}(D)$ with *n* ≥ 2.

*For any SU*(*2*) *matrix D*, *there exist SU*(*2*) *matrices A*, *B*, *and C such that ABC* = *I*_2_ *and AXBXC* = *D*, *and the gates* ${C}_{1}^{0}(D)$ *and* ${V}_{1}^{0}(D)$ *can be simulated by networks of the form shown in* (*a*) *and* (*b*) Fig. 6. *Here*, *SU*(*2*) *is the Lie group of* 2 × 2 *unitary matrices with determinant 1*.

More details of Lemmas 2, 3 and 4 are described in the reference^27^. Next, we derive the following corollaries.

#### **Corollary 1**.

*For any n* ≥ 7, $r=\lfloor n/2\rfloor$ *and m* ∈ {*r* + 2, *r* + 3, …, *n* − 2}, *an* ${N}_{n}^{m}$ *gate can be simulated by* 4(*m* − 1) *Toffoli gates and four basic operations*.

#### *Proof*.

Applying Lemma 2, an ${N}_{n}^{m}$ gate can be simulated by two ${N}_{n}^{r}$ gates and two ${N}_{n}^{m-r+1}$ gates. Noting that $3\le r\le \lfloor \frac{n}{2}\rfloor$ and $3\le m-r+1\le \lfloor \frac{n}{2}\rfloor$, we apply lemma 1 so that the corollary holds.$\square$

#### **Corollary 2**.

*For any n* ≥ 6 *and* $r=\lfloor n/2\rfloor$, *an* ${N}_{n}^{r+1}$ *gate can be simulated by* (4*r* − 2) *Toffoli gates and two basic operations when n is even*, *and* 4(*r* − 1) *Toffoli gates when n is odd*.

#### *Proof*.

When *n* is odd, $r+1=\lceil n/2\rceil$. Then, applying Lemma 3, we have that an ${N}_{n}^{r+1}$ gate can be simulated by a network consisting of 4(*r* − 1) Toffoli gates.

When *n* is even, by applying Lemma 2, it is derived that an ${N}_{n}^{r+1}$ gate can be simulated by two ${N}_{n}^{r}$ gates and two Toffoli gates. Then, by applying Lemma 1, it is proved that one can use (4*r* − 2) Toffoli gates and two basic operations to simulate the ${N}_{n}^{r+1}$ gate.$\square$

From lemma 4, the following corollary holds.

#### **Corollary 3**.

*For any SU*(*2*) *matrix D*, *there exist SU*(*2*) *matrices A*, *B*, *and C such that ABC* = *I*_2_ *and AXBXC* = *D*, *and the gates* ${C}_{n-1}^{0}(D)$ *and* ${V}_{n-1}^{0}(D)$ *can be simulated by networks of the form shown in* (*c*) *and* (*d*) *of* Fig. 6.

To analyze the complexities of the gates ${C}_{n-1}^{0}(X)$ and ${V}_{n-1}^{0}(X)$, we define three matrices:

12 $$\{\begin{array}{rcl}{R}_{z}(\theta ) & = & [\begin{array}{cc}{e}^{i\frac{\theta }{2}} & 0\\ 0 & {e}^{-i\frac{\theta }{2}}\end{array}],\\ {\rm{\Phi }}(\theta ) & = & [\begin{array}{cc}{e}^{i\theta } & 0\\ 0 & {e}^{i\theta }\end{array}],\\ {R}_{y}(\theta ) & = & [\begin{array}{cc}\cos \,\frac{\theta }{2} & \sin \,\frac{\theta }{2}\\ -\sin \,\frac{\theta }{2} & \cos \,\frac{\theta }{2}\end{array}].\end{array}$$

#### **Lemma 5**.

**Figure 7:**
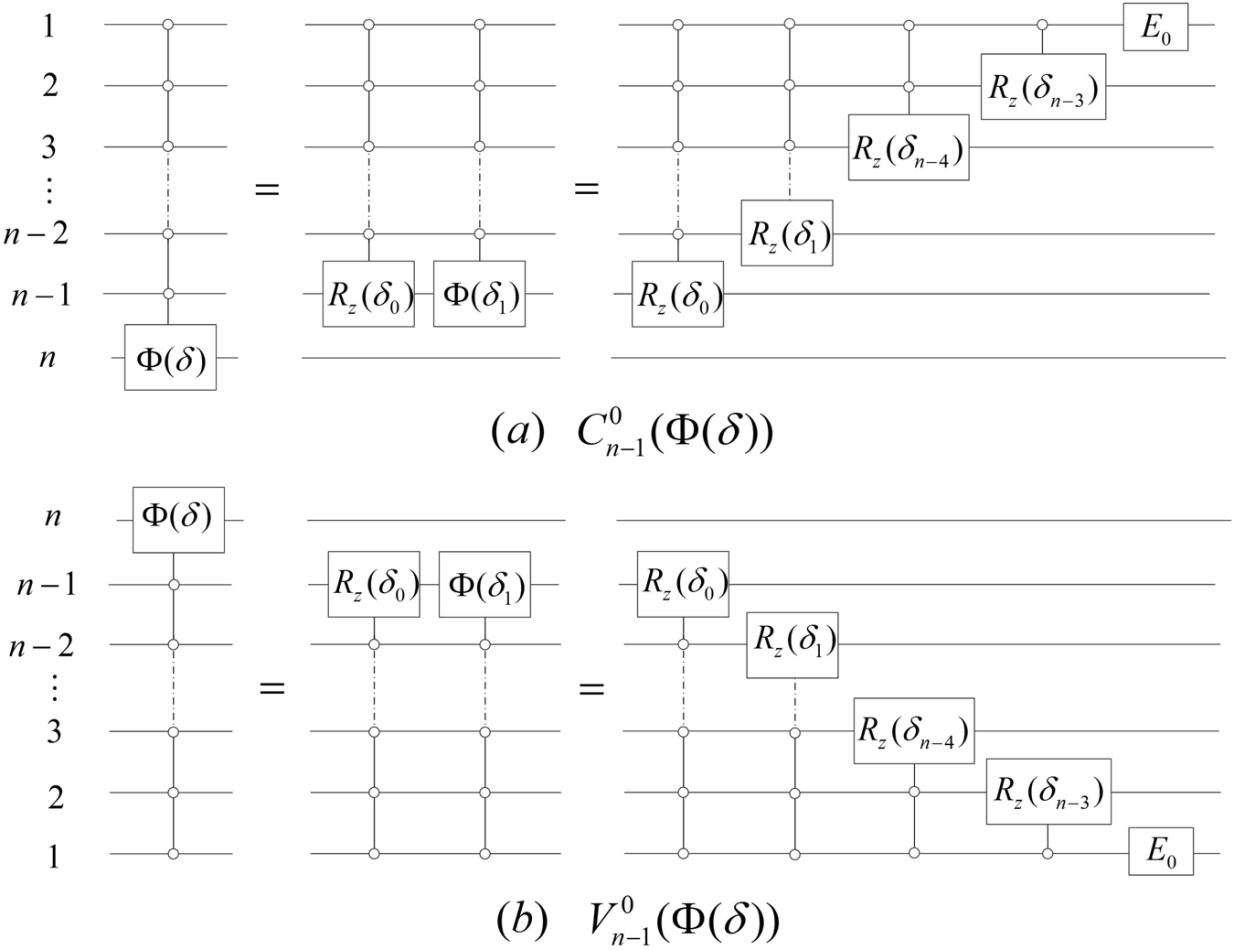
The simulated networks of the gates ${C}_{n-1}^{0}({\rm{\Phi }}(\delta ))$ and ${V}_{n-1}^{0}({\rm{\Phi }}(\delta ))$.

*Let* ${\delta }_{i}=\frac{\delta }{{2}^{i}}$, *i* ∈ {0, 1, 2, …, *n*}, *E*_0_ = Φ(*δ*_*n*−1_)*R*_*z*_(*δ*_*n*−2_). *Then*, *the gates* ${C}_{n-1}^{0}({\rm{\Phi }}(\delta ))$ *and* ${V}_{n-1}^{0}({\rm{\Phi }}(\delta ))$ *can be simulated by networks of the form shown in* Fig. 7.

#### *Proof*.

Note that

13 $${C}_{1}^{0}({\rm{\Phi }}(\delta ))=({\rm{\Phi }}(\frac{\delta }{2}){R}_{z}(\delta ))\otimes {I}_{2}=({\rm{\Phi }}({\delta }_{1}){R}_{z}({\delta }_{0}))\otimes {I}_{2},$$

14 $${V}_{1}^{0}({\rm{\Phi }}(\delta ))={I}_{2}\otimes ({\rm{\Phi }}(\frac{\delta }{2}){R}_{z}(\delta ))={I}_{2}\otimes ({\rm{\Phi }}({\delta }_{1}){R}_{z}({\delta }_{0})).$$

Then, we have that

15 $$\begin{array}{rcl}{C}_{n-1}^{0}({\rm{\Phi }}(\delta )) & = & {C}_{n-2}^{0}({C}_{1}^{0}({\rm{\Phi }}(\delta )))\\  & = & {C}_{n-2}^{0}(({\rm{\Phi }}({\delta }_{1}){R}_{z}({\delta }_{0}))\otimes {I}_{2})\\  & = & [{C}_{n-2}^{0}(({\rm{\Phi }}({\delta }_{1})){C}_{n-2}^{0}({R}_{z}({\delta }_{0}))]\otimes {I}_{2}.\end{array}$$

Similarly,

16 $${V}_{n-1}^{0}({\rm{\Phi }}(\delta ))={I}_{2}\otimes [{V}_{n-2}^{0}(({\rm{\Phi }}({\delta }_{1})){V}_{n-2}^{0}({R}_{z}({\delta }_{0}))].$$

Therefore, we have the simulated networks of the gates ${C}_{n-1}^{0}({\rm{\Phi }}(\delta ))$ and ${V}_{n-1}^{0}({\rm{\Phi }}(\delta ))$ as shown in Fig. 7.$\square$

#### **Lemma 6**.

**Figure 8:**
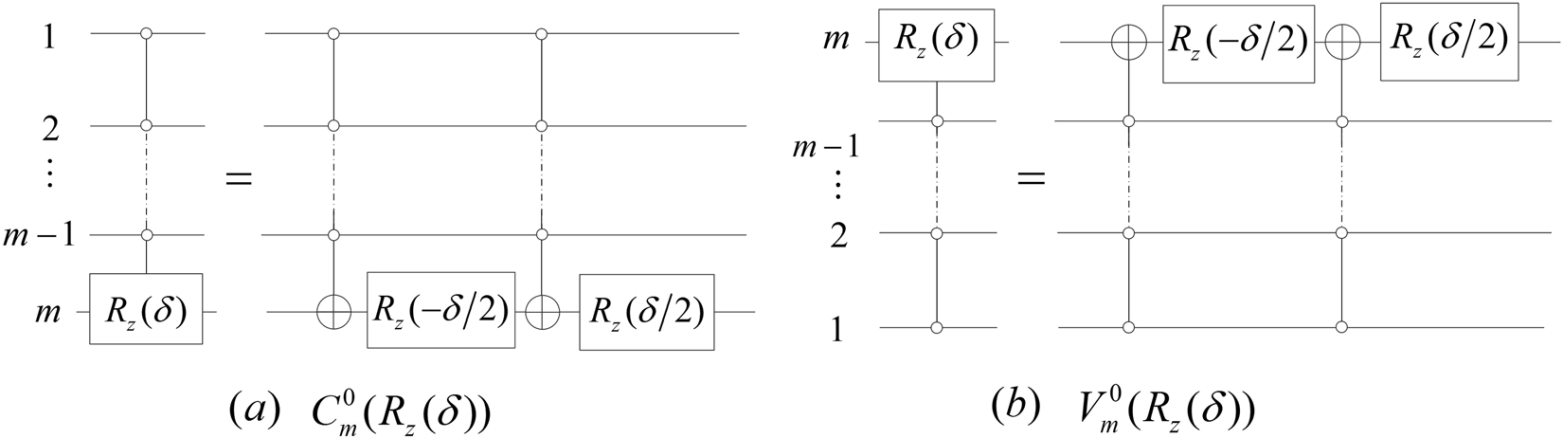
The simulated networks of the gates ${C}_{m}^{0}({R}_{z}(\delta ))$ and ${V}_{m}^{0}({R}_{z}(\delta ))$.

*The gates* ${C}_{m}^{0}({R}_{z}(\delta ))$ *and* ${V}_{m}^{0}({R}_{z}(\delta ))$ *can be simulated by networks of the form shown in* Fig. 8.

#### *Proof*.

Due to ${R}_{z}(\frac{\delta }{2}){R}_{z}(\,-\frac{\delta }{2})={I}_{2}$ and ${R}_{z}(\frac{\delta }{2})X{R}_{z}(\,-\frac{\delta }{2})X={R}_{z}(\delta )$, the conclusion is obvious.$\square$

**Figure 9:**
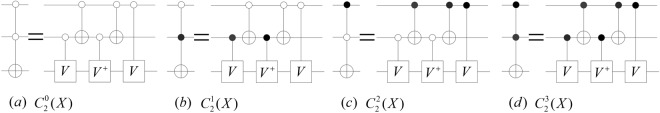
The simulated networks of the gates ${C}_{2}^{i}(X)$ (*i* = 0, 1, 2, 3).

${C}_{2}^{i}(X)$ (*i* = 0, 1, 2, 3) can be simulated by five basic operations shown in Fig. 9, i.e., $C({C}_{2}^{i}(X))={C}^{p}({C}_{2}^{i}(X))=5$.

Similarly, $C({V}_{2}^{i}(X))={C}^{p}({V}_{2}^{i}(X))=5$. Therefore, the complexity of Toffoli gates is 5. Thus, we obtain the complexity of ${C}_{n-1}^{0}(X)$ and ${V}_{n-1}^{0}(X)$ as described in theorem 1 below.

#### **Theorem 1**.

*For any n* ≥ 7, *the gates* ${C}_{n-1}^{0}(X)$ *and* ${V}_{n-1}^{0}(X)$ *can be simulated by* (3.5*n*^2^ − 13*n* − 4) *Toffoli gates and* 7*n* − 4 *basic operations when n is even*, *and by* (3.5*n*^2^ − 12*n* − 5.5) *Toffoli gates and* 7*n* − 3 *basic operations when n is odd*.

#### *Proof*.

Let *δ* = *π*/2, *D* = *R*_*z*_(−*π*)*R*_*y*_(*π*), *A* = *R*_*z*_(−*π*)*R*_*y*_(*π*), *B* = *R*_*y*_(−*π*/2)*R*_*z*_(*π*/2) and *C* = *R*_*z*_(*π*/2). Then, *D*, *A*, *B*, *C* ∈ *SU*(2), *ABC* = *I*_2_, *AXBXC* = *D* and Φ(*δ*)*D* = *X*.

Note that ${C}_{n-1}^{0}(X)={C}_{n-1}^{0}({\rm{\Phi }}(\delta )){C}_{n-1}^{0}(D)$. Then,

17 $$C({C}_{n-1}^{0}(X))=C({C}_{n-1}^{0}({\rm{\Phi }}(\delta )))+C({C}_{n-1}^{0}(D)).$$

From lemma 4 and corollary 3, we obtain

18 $$C({C}_{n-1}^{0}(D))=2C({N}_{n}^{n-2})+15.$$

By lemma 5 and lemma 6, $C({C}_{n-1}^{0}({\rm{\Phi }}(\delta )))$ can be computed by

19 $$C({C}_{n-1}^{0}({\rm{\Phi }}(\delta )))=\sum _{i=1}^{n-2}\,C({C}_{i}^{0}({R}_{z}({\delta }_{n-2-i})))+1=2\,\sum _{i=1}^{n-2}\,C({N}_{n}^{i})+2n-3.$$

Therefore,

20 $$C({C}_{n-1}^{0}(X))=\sum _{i=3}^{r}\,2C({N}_{n}^{i})+\sum _{i=r+2}^{n-3}\,2C({N}_{n}^{i})+4C({N}_{n}^{n-2})+2C({N}_{n}^{r+1})+2C({N}_{3}^{2})+2n+14,$$

where $r=\lfloor n/2\rfloor$ and ${N}_{3}^{2}$ is a Toffoli gate.

Applying lemma 1, corollary 1 and corollary 2, we obtain

21 $$C({C}_{n-1}^{0}(X))=\{\begin{array}{l}(3.5{n}^{2}-13n-4)C({N}_{3}^{2})+7n-4,\,{\rm{when}}\,{\rm{n}}\,{\rm{is}}\,{\rm{even}},\\ (3.5{n}^{2}-12n-5.5)C({N}_{3}^{2})+7n-3,\,{\rm{when}}\,{\rm{n}}\,{\rm{is}}\,{\rm{odd}}.\end{array}$$

Similarly, we obtain that $C({V}_{n-1}^{0}(X))=C({C}_{n-1}^{0}(X))$.$\square$

Comparing with the methods proposed in^27^, the complexities of our proposed simulated networks of these gates ${N}_{n}^{i}\mathrm{(3}\le i\le n-\mathrm{1)}$ are reduced by 50% approximately.

## The Quantum Circuits of PSP

**Figure 10:**
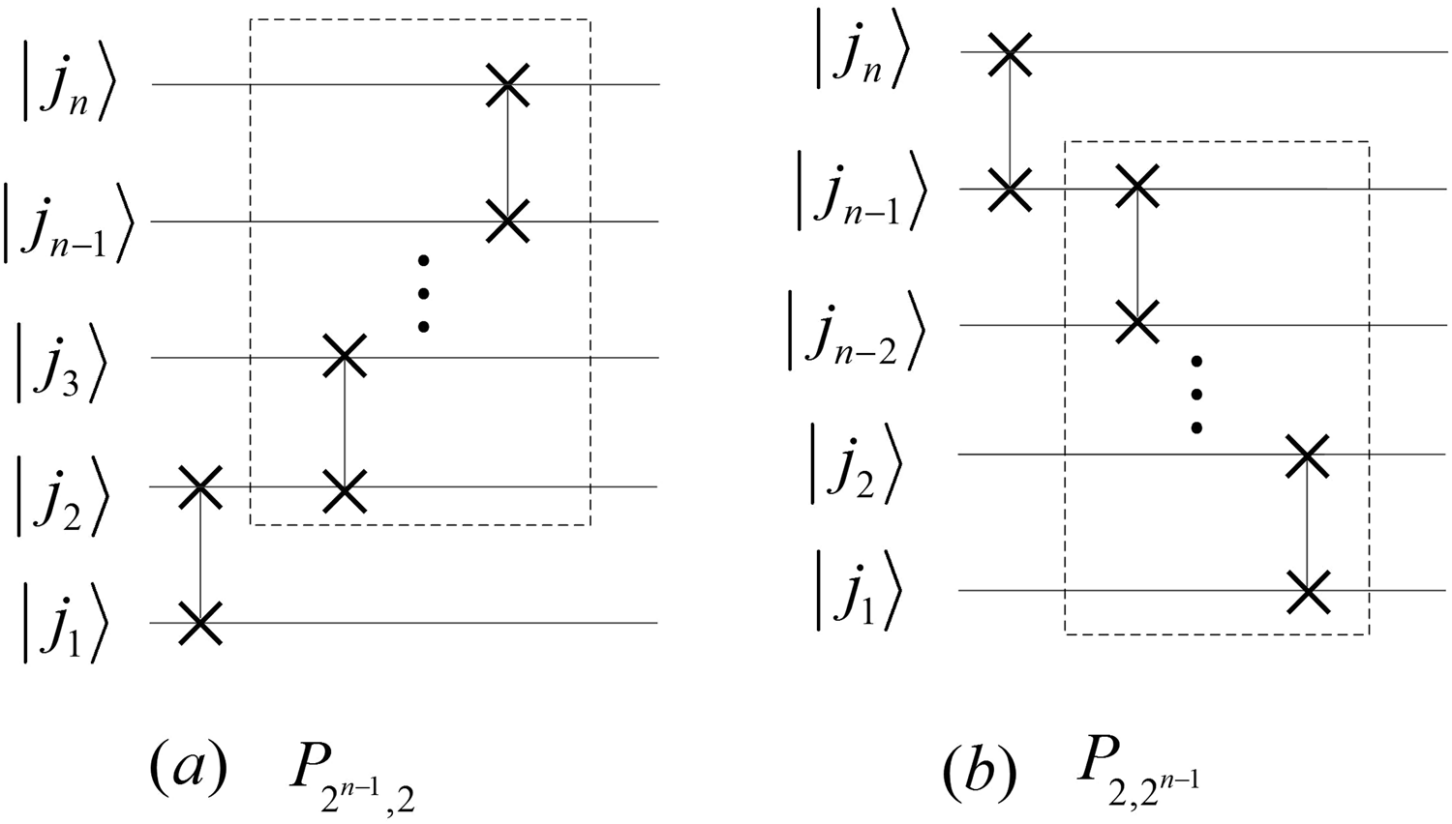
The implement circuits of ${P}_{{2}^{n-1}\mathrm{,2}}$ and ${P}_{{\mathrm{2,2}}^{n-1}}$. The dotted boxes in (**a**,**b**) are the circuits of ${P}_{{2}^{n-2}\mathrm{,2}}$ and ${P}_{{\mathrm{2,2}}^{n-2}}$, respectively.

The perfect shuffle permutation ${P}_{{2}^{n-1}\mathrm{,2}}$ and ${P}_{{\mathrm{2,2}}^{n-1}}$ can be expressed as

22 $$\{\begin{array}{l}{P}_{{2}^{n-1},2}=({P}_{{2}^{n-2},2}\otimes {I}_{2})\,({I}_{{2}^{n-2}}\otimes {P}_{2,2}),\\ {P}_{2,{2}^{n-1}}=({I}_{2}\otimes {P}_{2,{2}^{n-2}})\,({P}_{2,2}\otimes {I}_{{2}^{n-2}}),\end{array}$$

where *P*_2,2_ is a Swap gate, and their implementation circuits are shown in Fig. 10.

Applying ${P}_{{2}^{n-1}\mathrm{,2}}$ and ${P}_{{\mathrm{2,2}}^{n-1}}$ to the state $|{j}_{n}{j}_{n-1}\cdots {j}_{2}{j}_{1}\rangle$, we have

23 $$\{\begin{array}{l}{P}_{{2}^{n-1},2}|{j}_{n}{j}_{n-1}\cdots {j}_{2}{j}_{1}\rangle =|{j}_{1}{j}_{n}{j}_{n-1}\cdots {j}_{2}\rangle ,\\ {P}_{2,{2}^{n-1}}|{j}_{n}{j}_{n-1}\cdots {j}_{2}{j}_{1}\rangle =|{j}_{n-1}\cdots {j}_{2}{j}_{1}{j}_{n}\rangle .\end{array}$$

Let ${{\rm{\Gamma }}}_{{2}^{n}}={P}_{{2}^{n-1},2}({P}_{{2}^{n-2},2}\otimes {I}_{2})\ldots ({P}_{{2}^{2},2}\otimes {I}_{{2}^{n-3}})\,({P}_{2,2}\otimes {I}_{{2}^{n-2}})$, we have that ${({{\rm{\Gamma }}}_{{2}^{n}})}^{-1}=({P}_{2,2}\otimes {I}_{{2}^{n-2}})$ $({P}_{2,{2}^{2}}\otimes {I}_{{2}^{n-3}})\ldots ({P}_{2,{2}^{n-2}}\otimes {I}_{2}){P}_{2,{2}^{n-1}}$ and

24 $${{\rm{\Gamma }}}_{{2}^{n}}|{j}_{n}{j}_{n-1}{j}_{n-2}\cdots {j}_{2}{j}_{1}\rangle =|{j}_{1}{j}_{2}\cdots {j}_{n-2}{j}_{n-1}{j}_{n}\rangle ={({{\rm{\Gamma }}}_{{2}^{n}})}^{-1}|{j}_{n}{j}_{n-1}{j}_{n-2}\cdots {j}_{2}{j}_{1}\rangle $$

**Figure 11:**
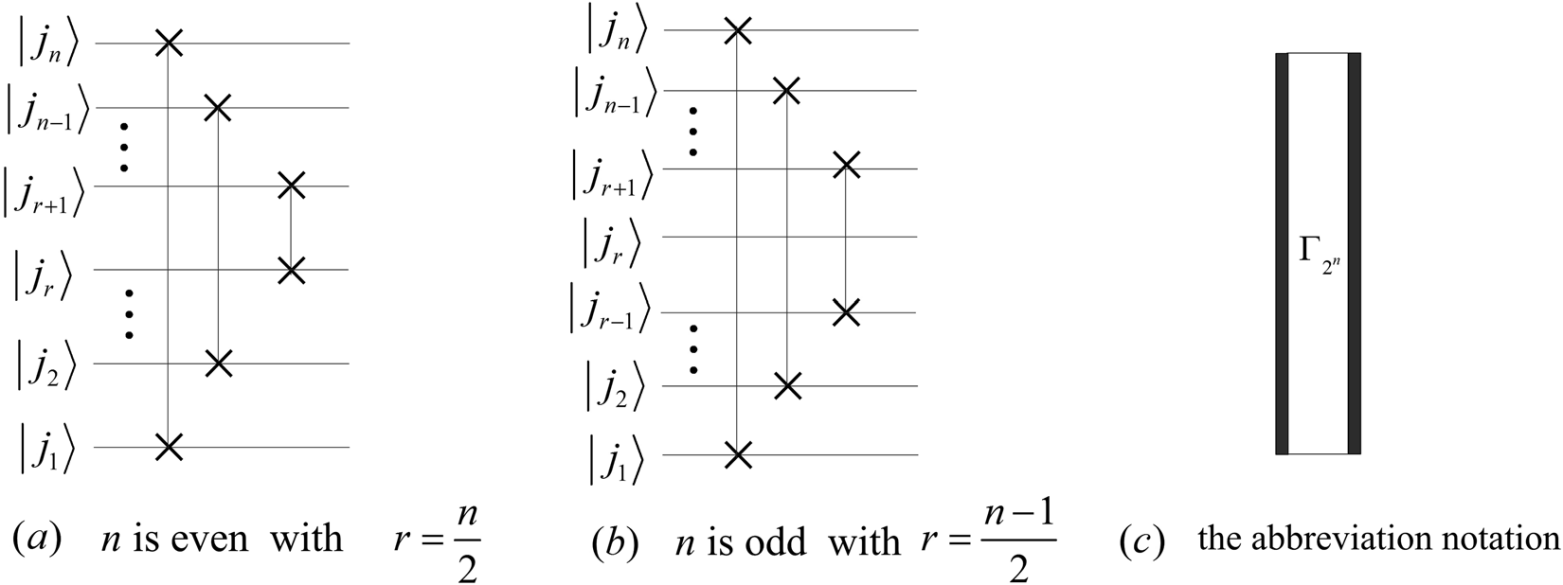
The simplified quantum circuits of ${{\rm{\Gamma }}}_{{2}^{n}}$ and ${({{\rm{\Gamma }}}_{{2}^{n}})}^{-1}$.

Therefore, we conclude that ${{\rm{\Gamma }}}_{{2}^{n}}={({{\rm{\Gamma }}}_{{2}^{n}})}^{-1}$ and design quantum circuits shown in Fig. 11.

The costs of the circuits of ${{\rm{\Gamma }}}_{{2}^{n}}$ and ${({{\rm{\Gamma }}}_{{2}^{n}})}^{-1}$ are

25 $$C({{\rm{\Gamma }}}_{{2}^{n}})=C({({{\rm{\Gamma }}}_{{2}^{n}})}^{-1})=3\times \lfloor \frac{n}{2}\rfloor .$$

**Figure 12:**
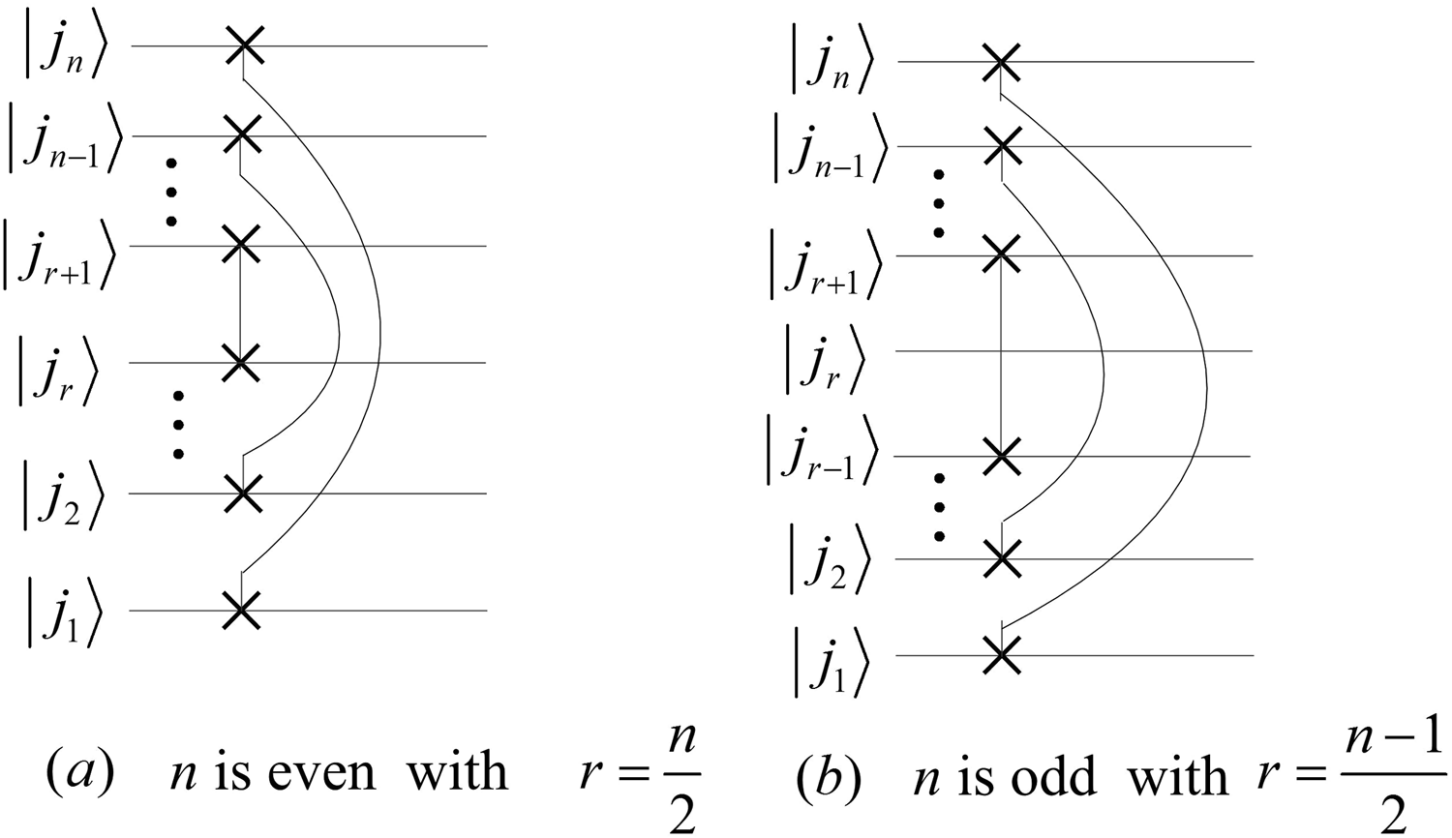
The parallel quantum circuits of ${{\rm{\Gamma }}}_{{2}^{n}}$ and ${({{\rm{\Gamma }}}_{{2}^{n}})}^{-1}$.

By parallel computing, we redesign the circuits of ${{\rm{\Gamma }}}_{{2}^{n}}$ and ${({{\rm{\Gamma }}}_{{2}^{n}})}^{-1}$ shown in Fig. 12 and calculate time complexities by

26 $${C}^{p}({{\rm{\Gamma }}}_{{2}^{n}})={C}^{p}({({{\rm{\Gamma }}}_{{2}^{n}})}^{-1})=C(Swap)=3.$$

i.e., complexities of ${{\rm{\Gamma }}}_{{2}^{n}}$ and ${({{\rm{\Gamma }}}_{{2}^{n}})}^{-1}$ are *O*(1).

The iterations of ${{\rm{\Gamma }}}_{{2}^{n}}$ and ${({{\rm{\Gamma }}}_{{2}^{n}})}^{-1}$ are given by

27 $$\{\begin{array}{rcl}{{\rm{\Gamma }}}_{{2}^{n}} & = & {P}_{{2}^{n-1},2}({{\rm{\Gamma }}}_{{2}^{n-1}}\otimes {I}_{2}),\\ {({{\rm{\Gamma }}}_{{2}^{n}})}^{-1} & = & ({({{\rm{\Gamma }}}_{{2}^{n-1}})}^{-1}\otimes {I}_{2}){P}_{2,{2}^{n-1}}.\end{array}$$

Then, we obtain

28 $$\{\begin{array}{lcc}{P}_{{2}^{n-1},2} & = & {{\rm{\Gamma }}}_{{2}^{n}}({{\rm{\Gamma }}}_{{2}^{n-1}}\otimes {I}_{2}),\\ {P}_{2,{2}^{n-1}} & = & ({{\rm{\Gamma }}}_{{2}^{n-1}}\otimes {I}_{2}){{\rm{\Gamma }}}_{{2}^{n}}.\end{array}$$

**Figure 13:**
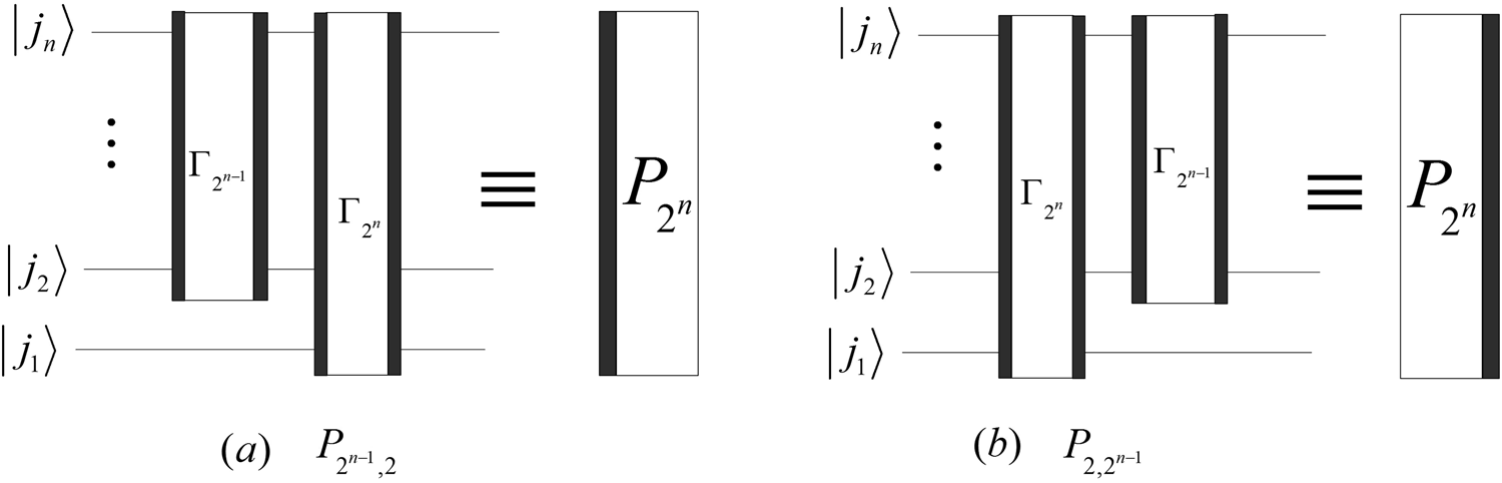
The simplified circuits of ${P}_{{2}^{n-1}\mathrm{,2}}$ and ${P}_{{\mathrm{2,2}}^{n-1}}$. The rights of (**a**,**b**) correspond to the abbreviation notation of ${P}_{{2}^{n-1}\mathrm{,2}}$ and ${P}_{{\mathrm{2,2}}^{n-1}}$, respectively.

Therefore, we design the simplified circuits of ${P}_{{2}^{n-1}\mathrm{,2}}$ and ${P}_{{\mathrm{2,2}}^{n-1}}$ as shown in Fig. 13.

The complexities of ${P}_{{2}^{n-1}\mathrm{,2}}$ and ${P}_{{\mathrm{2,2}}^{n-1}}$ are

29 $$\{\begin{array}{rcl}C({P}_{{2}^{n-1},2}) & = & C({P}_{2,{2}^{n-1}})=3(n-1),\\ {C}^{P}({P}_{{2}^{n-1},2}) & = & {C}^{P}({P}_{2,{2}^{n-1}})=6.\end{array}$$

The reason that the abbreviation notations in Fig. 13 are the same except for the positions of black boxes is due to the fact that the circuit in Fig. 13(b) consists of the gates in Fig. 13(a) but rearranged in reverse order. We also adopt similar abbreviation notations to denote the circuits that are composed of the same quantum gates with reverse order in the following sections.

The iterations of ${P}_{{2}^{n}{\mathrm{,2}}^{m-1}}$ and ${P}_{{2}^{m-1}{\mathrm{,2}}^{n}}$ are given by

30 $$\{\begin{array}{l}{P}_{{2}^{n},{2}^{m-1}}=({P}_{2,{2}^{m-1}}\otimes {I}_{{2}^{n-1}})\,({I}_{2}\otimes {P}_{{2}^{n-1},{2}^{m-1}}),\\ {P}_{{2}^{m-1},{2}^{n}}=({I}_{2}\otimes {P}_{{2}^{m-1},{2}^{n-1}})\,({P}_{{2}^{m-1},2}\otimes {I}_{{2}^{n-1}}).\end{array}$$

**Figure 14:**
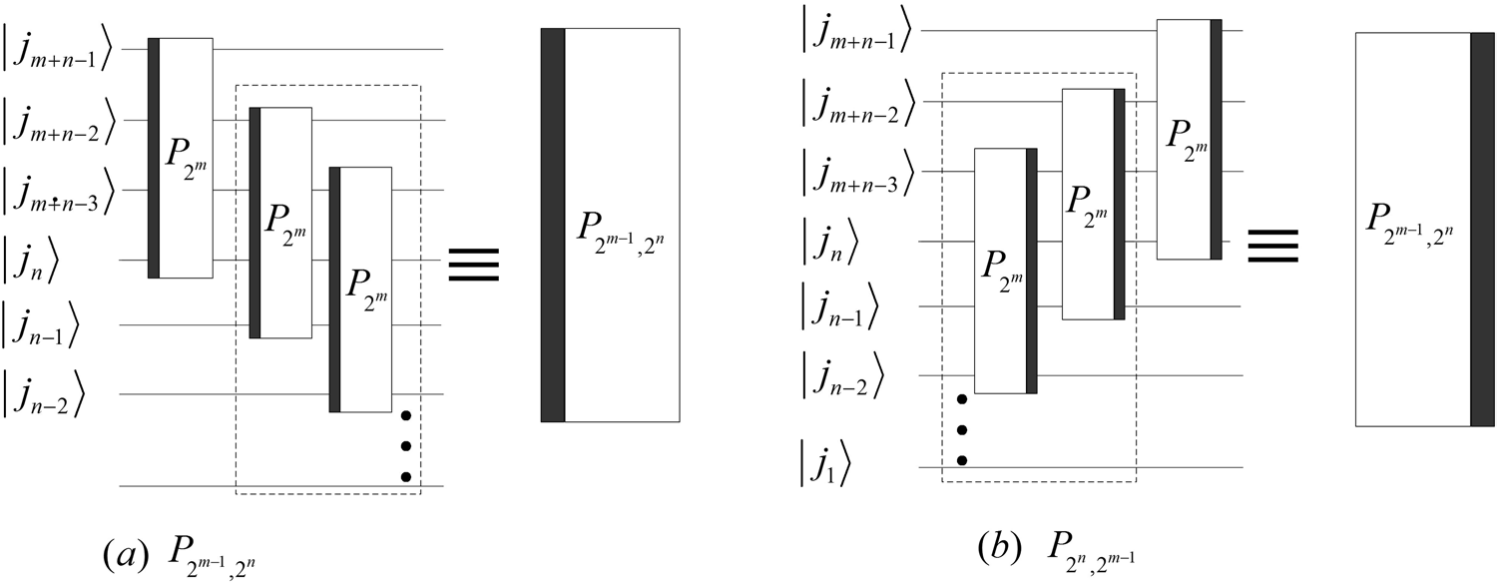
The quantum circuits of ${P}_{{2}^{m-1}{\mathrm{,2}}^{n}}$ and ${P}_{{2}^{n}{\mathrm{,2}}^{m-1}}$. The dotted boxes in (**a**,**b**) are the implement circuits of ${P}_{{2}^{m-1}{\mathrm{,2}}^{n-1}}$ and ${P}_{{2}^{n-1}{\mathrm{,2}}^{m-1}}$, respectively.

According to (30), we design the implementation circuits of ${P}_{{2}^{m-1}{\mathrm{,2}}^{n}}$ and ${P}_{{2}^{n}{\mathrm{,2}}^{m-1}}$ in Fig. 14.

The complicities of the circuits in Fig. 14 are

31 $$\{\begin{array}{rcl}C({P}_{{2}^{m-1},{2}^{n}}) & = & C({P}_{{2}^{n},{2}^{m-1}})=3n(m-1),\\ {C}^{p}({P}_{{2}^{m-1},{2}^{n}}) & = & {C}^{p}({P}_{{2}^{n},{2}^{m-1}})=6n.\end{array}$$

## The Implementation of QWPT

Let ${W}_{{2}^{n}}^{0}={W}_{{2}^{n}}$ be a wavelet kernel matrix. Then, the (*k* + 1)-th iteration of a discrete wavelet packet transform is defined by

32 $$\{\begin{array}{l}{Z}_{{2}^{n}}^{k}={W}_{{2}^{n}}^{k}{W}_{{2}^{n}}^{k-1}\ldots {W}_{{2}^{n}}^{1}{W}_{{2}^{n}}^{0},\\ {W}_{{2}^{n}}^{j}=Diag({W}_{{2}^{n-j}},{W}_{{2}^{n-j}},\ldots ,{W}_{{2}^{n-j}}),\end{array}$$

where *j* = 1, …, *k* and $Diag({W}_{{2}^{n-j}},{W}_{{2}^{n-j}},\ldots ,{W}_{{2}^{n-j}})$ is a matrix with 2^*j*^ blocks of ${W}_{{2}^{n-j}}$ on the main diagonal and zeros elsewhere.

The following equations

33 $$\{\begin{array}{l}{W}_{{2}^{n}}^{j}=Diag({W}_{{2}^{n-1}}^{j-1},{W}_{{2}^{n-1}}^{j-1}),\\ {Z}_{{2}^{n}}^{k}=Diag({Z}_{{2}^{n-1}}^{k-1},{Z}_{{2}^{n-1}}^{k-1}){W}_{{2}^{n}}\end{array}$$

can be derived by (32).

**Figure 15:**
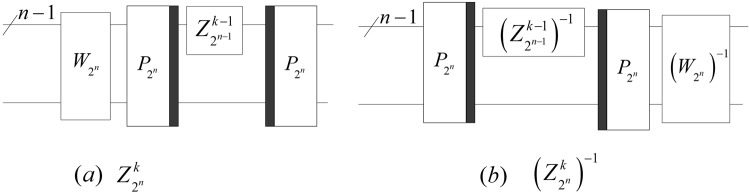
The implementation circuits of ${Z}_{{2}^{n}}^{k}$ and ${({Z}_{{2}^{n}}^{k})}^{-1}$.

Since

34 $$\begin{array}{l}{Z}_{{2}^{n-1}}^{k-1}\otimes {I}_{2}={P}_{2,{2}^{n-1}}Diag({Z}_{{2}^{n-1}}^{k-1},{Z}_{{2}^{n-1}}^{k-1}){P}_{{2}^{n-1},2},\end{array}$$

the iteration equation of the QWPT is given by

35 $${Z}_{{2}^{n}}^{k}={P}_{{2}^{n-1},2}({Z}_{{2}^{n-1}}^{k-1}\otimes {I}_{2}){P}_{2,{2}^{n}}{W}_{{2}^{n}}$$

with the initial value ${Z}_{{2}^{n-k}}^{0}={W}_{{2}^{n-k}}$ and the implementation circuit shown in (a) of Fig. 15.

Similarly, the inverse of ${Z}_{{2}^{n}}^{k}$ is

36 $${({Z}_{{2}^{n}}^{k})}^{-1}={({W}_{{2}^{n}})}^{-1}{P}_{{2}^{n-1},2}({({Z}_{{2}^{n-1}}^{k-1})}^{-1}\otimes {I}_{2}){P}_{2,{2}^{n-1}}$$

with the initial value ${({Z}_{{2}^{n-k}}^{0})}^{-1}={({W}_{{2}^{n-k}})}^{-1}$ and the implementation circuit of ${({Z}_{{2}^{n}}^{k})}^{-1}$ shown in (b) of Fig. 15.

Next, we describe the implementations of the Haar QWPT (HQWPT) and the D4 QWPT (DQWPT) in detail.

### The implementation of HQWPT

Substituting the kernel matrix ${W}_{{2}^{n}}={P}_{{2}^{n-1},2}({I}_{{2}^{n-1}}\otimes H)$ into equations (35) and (36), the (*k* + 1)-th iteration of HQWPT and its inverse are

37 $$\{\begin{array}{rcl}{R}_{{2}^{n}}^{k} & = & {P}_{{2}^{n-1},2}({R}_{{2}^{n-1}}^{k-1}\otimes {I}_{2})\,({I}_{{2}^{n-1}}\otimes H),\\ {({R}_{{2}^{n}}^{k})}^{-1} & = & ({I}_{{2}^{n-1}}\otimes H)\,({({R}_{{2}^{n-1}}^{k-1})}^{-1}\otimes {I}_{2}){P}_{2,{2}^{n-1}}\end{array}$$

with the initial values

$$\{\begin{array}{rcl}{R}_{{2}^{n-k}}^{0} & = & {P}_{{2}^{n-k-1},2}({I}_{{2}^{n-k-1}}\otimes H),1\le k < n-1,\\ {R}_{2}^{0} & = & H,k=n-1,\\ {({R}_{{2}^{n-k}}^{0})}^{-1} & = & (H\otimes {I}_{{2}^{n-k-1}}){P}_{2,{2}^{n-k-1}},1\le k < n-1,\\ {({R}_{2}^{0})}^{-1} & = & H,k=n-1.\end{array}$$

**Figure 16:**
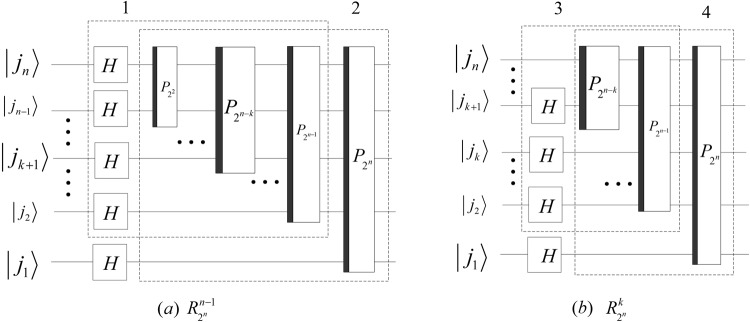
The implementation circuits of ${R}_{{2}^{n}}^{n-1}$ and ${R}_{{2}^{n}}^{k}$ (1 ≤ *k* < *n* − 1). The dashed box 1 and box 3 implement ${R}_{{2}^{n-1}}^{n-2}$ and ${R}_{{2}^{n-1}}^{k-1}$, respectively.

The quantum circuits of ${R}_{{2}^{n}}^{n-1}$ and ${R}_{{2}^{n}}^{k}$ (1 ≤ *k* < *n* − 1) are designed in Fig. 16.

**Figure 17:**
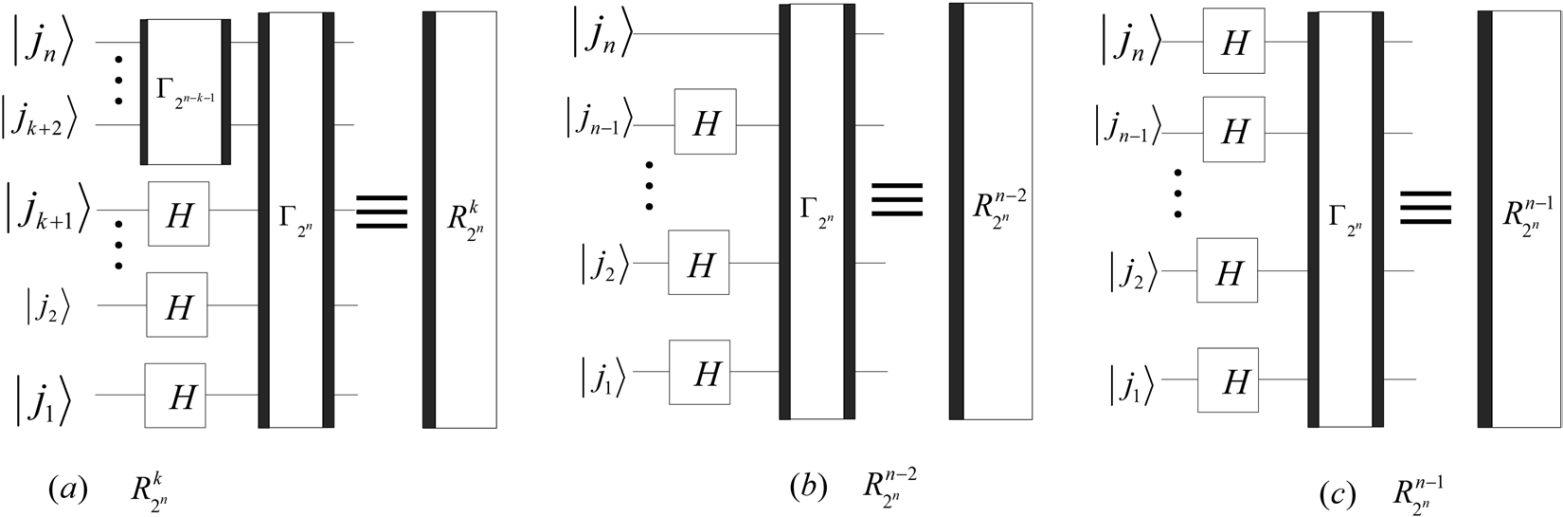
The simplified circuits of ${R}_{{2}^{n}}^{k}$ 1 ≤ *k* < *n* − 2, ${R}_{{2}^{n}}^{n-2}$ and ${R}_{{2}^{n}}^{n-1}$.

Since ${P}_{{2}^{n-1},2}({P}_{{2}^{n-2},2}\otimes {I}_{2})\,\ldots \,({P}_{2,2}\otimes {I}_{{2}^{n-2}})={{\rm{\Gamma }}}_{{2}^{n}}$ and ${P}_{{2}^{n-1},2}({P}_{{2}^{n-2},2}\otimes {I}_{2})\,\ldots \,({P}_{{2}^{n-k-1},2}\otimes {I}_{{2}^{k}})={{\rm{\Gamma }}}_{{2}^{n}}({({{\rm{\Gamma }}}_{{2}^{n-k-1}})}^{-1}$ $\otimes {I}_{{2}^{k+1}})$ with 1 ≤ *k* < *n* − 2, the quantum circuit of ${R}_{{2}^{n}}^{k}$, ${R}_{{2}^{n}}^{n-2}$ and ${R}_{{2}^{n}}^{n-1}$ can be simplified and shown in Fig. 17.

**Figure 18:**
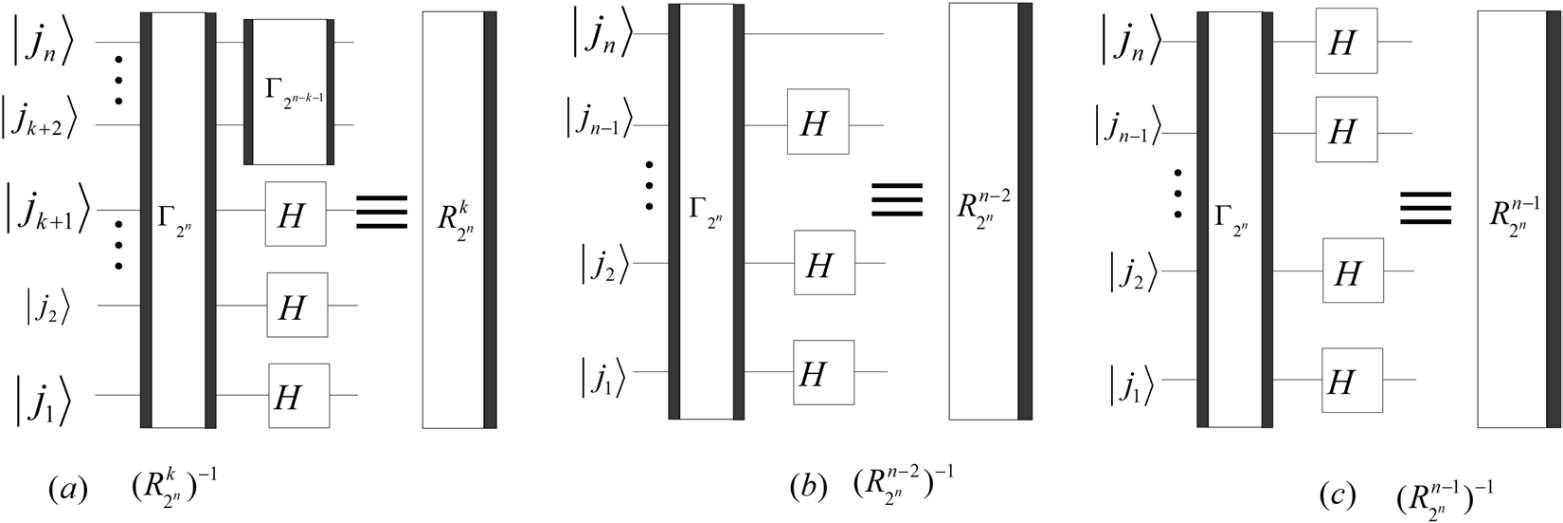
The simplified circuits of ${({R}_{{2}^{n}}^{k})}^{-1}$ 1 ≤ *k* < *n* − 2, ${({R}_{{2}^{n}}^{n-2})}^{-1}$ and ${({R}_{{2}^{n}}^{n-1})}^{-1}$.

Similarly, the quantum circuits of the inverses of ${R}_{{2}^{n}}^{k}$, ${R}_{{2}^{n}}^{n-2}$ and ${R}_{{2}^{n}}^{n-1}$ can be designed as shown in Fig. 18.

The costs of HQWPT are

38 $$\{\begin{array}{rcl}C({R}_{{2}^{n}}^{k}) & = & C({({R}_{{2}^{n}}^{k})}^{-1})=3\lfloor \frac{n}{2}\rfloor +3\lfloor \frac{n\,-\,k\,-\,1}{2}\rfloor +k+1,\\ C({R}_{{2}^{n}}^{n-2}) & = & C({({R}_{{2}^{n}}^{n-2})}^{-1})=3\lfloor \frac{n}{2}\rfloor +n-1,\\ C({R}_{{2}^{n}}^{n-1}) & = & C({({R}_{{2}^{n}}^{n-1})}^{-1})=3\lfloor \frac{n}{2}\rfloor +n,\end{array}$$

where 1 ≤ *k* < *n* − 2. Since ${C}^{p}({R}_{{2}^{n}}^{k})={C}^{p}({({R}_{{2}^{n}}^{k})}^{-1})=6,1\le k < n-2$ and ${C}^{p}({R}_{{2}^{n}}^{k})={C}^{p}({({R}_{{2}^{n}}^{k})}^{-1})=4,n-2\le$ $k\le n-1$, the time complexity of the HQWPT is *O*(1).

### The implementation of DQWPT

The kernel matrix of the D4 wavelet transform is defined by the reference^31^

39 $${D}_{{2}^{n}}=[\begin{array}{lllllllllll}{h}_{0} & {h}_{1} & {h}_{2} & {h}_{3} & 0 & 0 & \cdots  & 0 & 0 & 0 & 0\\ {h}_{3} & -{h}_{2} & {h}_{1} & -{h}_{0} & 0 & 0 & \cdots  & 0 & 0 & 0 & 0\\ 0 & 0 & {h}_{0} & {h}_{1} & {h}_{2} & {h}_{3} & \cdots  & 0 & 0 & 0 & 0\\ 0 & 0 & {h}_{3} & -{h}_{2} & {h}_{1} & -{h}_{0} & \cdots  & 0 & 0 & 0 & 0\\ \vdots  & \vdots  & \vdots  & \vdots  & \vdots  & \vdots  & \ddots  & \vdots  & \vdots  & \vdots  & \vdots \\ 0 & 0 & 0 & 0 & 0 & 0 & \cdots  & {h}_{0} & {h}_{1} & {h}_{2} & {h}_{3}\\ 0 & 0 & 0 & 0 & 0 & 0 & \cdots  & {h}_{3} & -{h}_{2} & {h}_{1} & -{h}_{0}\\ {h}_{2} & {h}_{3} & 0 & 0 & 0 & 0 & \cdots  & 0 & 0 & {h}_{0} & {h}_{1}\\ {h}_{1} & -{h}_{0} & 0 & 0 & 0 & 0 & \cdots  & 0 & 0 & {h}_{3} & -{h}_{2}\end{array}],$$

where $${h}_{0}=\frac{1+\sqrt{3}}{4\sqrt{2}}$$, $${h}_{1}=\frac{3+\sqrt{3}}{4\sqrt{2}}$$, $${h}_{2}=\frac{3-\sqrt{3}}{4\sqrt{2}}$$ and $${h}_{3}=\frac{1-\sqrt{3}}{4\sqrt{2}}$$.

**Figure 19:**
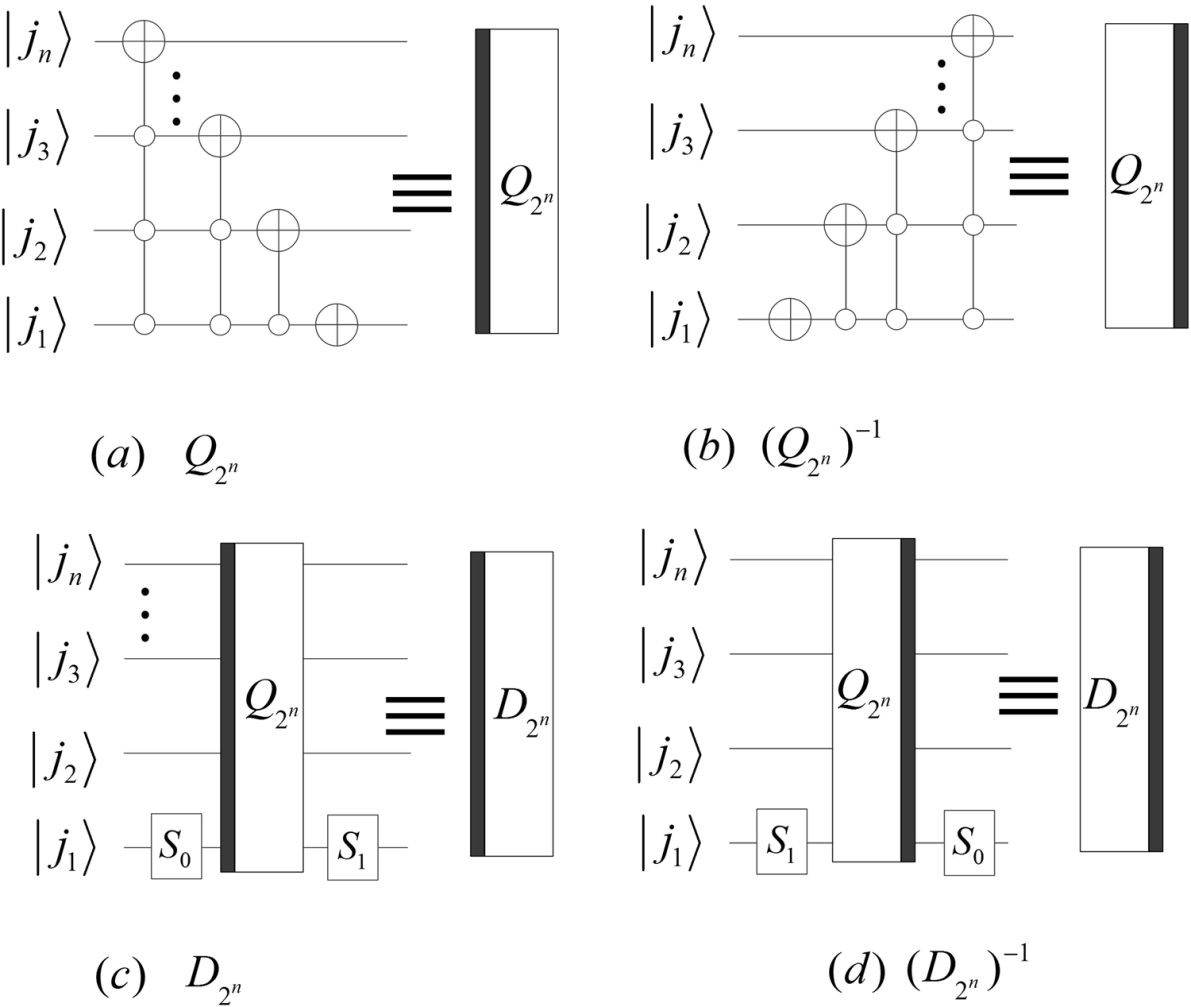
The quantum circuits of the kernel matrix of the D4 wavelet transform.

${D}_{{2}^{n}}$ and ${({D}_{{2}^{n}})}^{-1}$ can be rewritten to

40 $$\{\begin{array}{rcl}{D}_{{2}^{n}} & = & ({I}_{{2}^{n-1}}\otimes {S}_{1}){Q}_{{2}^{n}}({I}_{{2}^{n-1}}\otimes {S}_{0}),\\ {Q}_{{2}^{n}} & = & (\{{Q}_{{2}^{n-1}},{I}_{{2}^{n-1}}\}\otimes {I}_{2}),\\ {({D}_{{2}^{n}})}^{-1} & = & ({I}_{{2}^{n-1}}\otimes {S}_{0})\,{({Q}_{{2}^{n}})}^{-1}({I}_{{2}^{n-1}}\otimes {S}_{1}),\\ {({Q}_{{2}^{n}})}^{-1} & = & (\{{({Q}_{{2}^{n-1}})}^{-1},{I}_{{2}^{n-1}}\}\otimes {I}_{2})\,({I}_{{2}^{n-1}}\otimes X),\end{array}$$

where

$$\{\begin{array}{rcl}{S}_{0} & = & [\begin{array}{ll}\sin (2\pi /3) & \cos (2\pi /3)\\ \cos (2\pi /3) & -\sin (2\pi /3)\end{array}]\\ {S}_{1} & = & [\begin{array}{ll}-\cos (\pi /12) & \sin (\pi /12)\\ \sin (\pi /12) & \cos (\pi /12)\end{array}]\\ {Q}_{2} & = & {({Q}_{2})}^{-1}=X,\end{array}$$

and the implementation circuits shown in Fig. 19.

In order to implement a multi-level DQWPT based on the periodization extension, a single-level DQWPT and its inverse are given by:

41 $$\{\begin{array}{rcl}{T}_{{2}^{n}} & = & {P}_{{2}^{n-1},2}{D}_{{2}^{n}}^{p},\\ {D}_{{2}^{n}}^{p} & = & {D}_{{2}^{n}}{({Q}_{{2}^{n}})}^{-1},\\ {({T}_{{2}^{n}})}^{-1} & = & {({D}_{{2}^{n}}^{p})}^{-1}{P}_{2,{2}^{n-1}},\\ {({D}_{{2}^{n}}^{p})}^{-1} & = & {Q}_{{2}^{n}}{({D}_{{2}^{n}})}^{-1}.\end{array}$$

**Figure 20:**
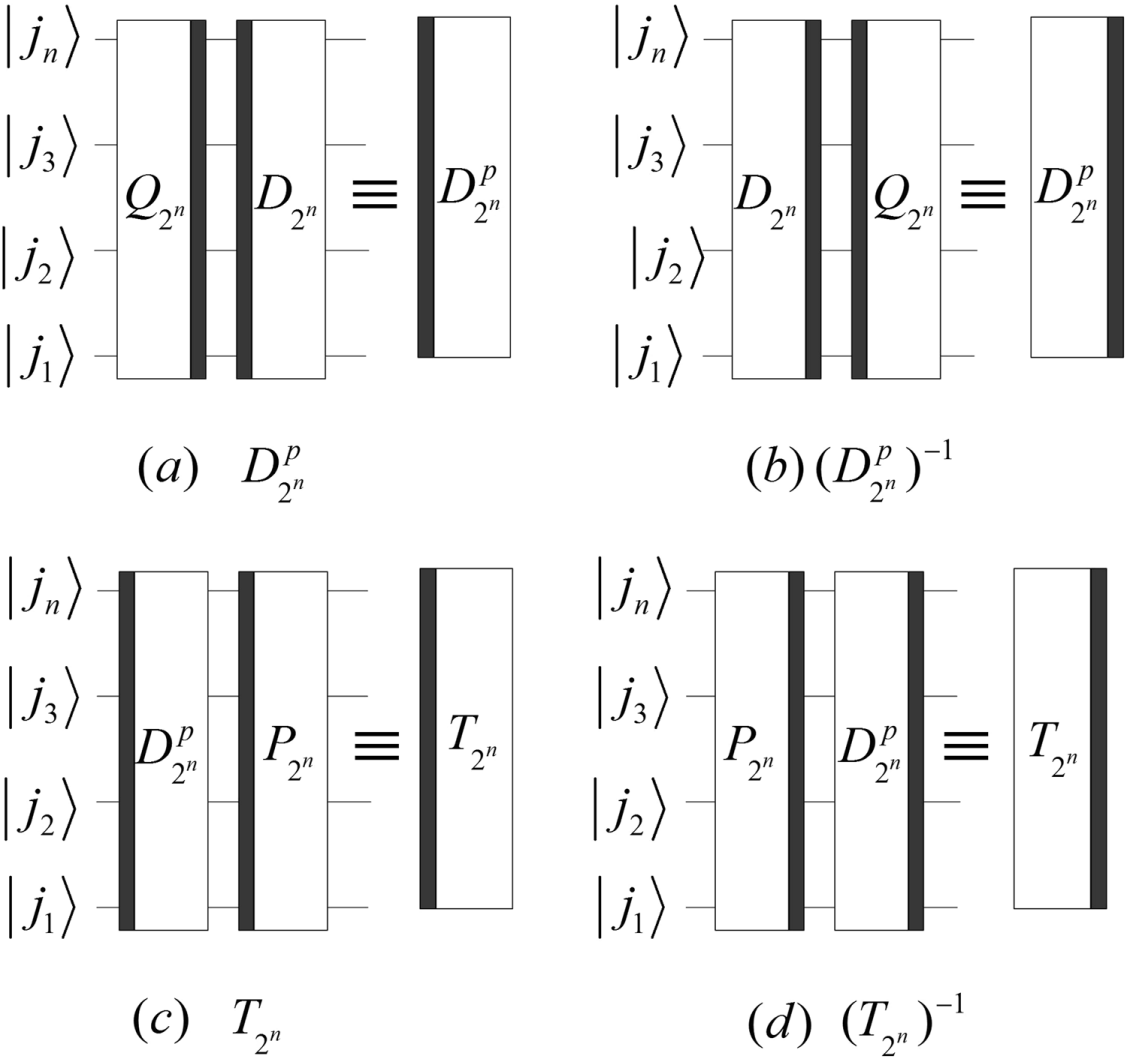
The quantum circuits of the single-level DQWPT and its inverse based on the periodization extension.

**Figure 21:**
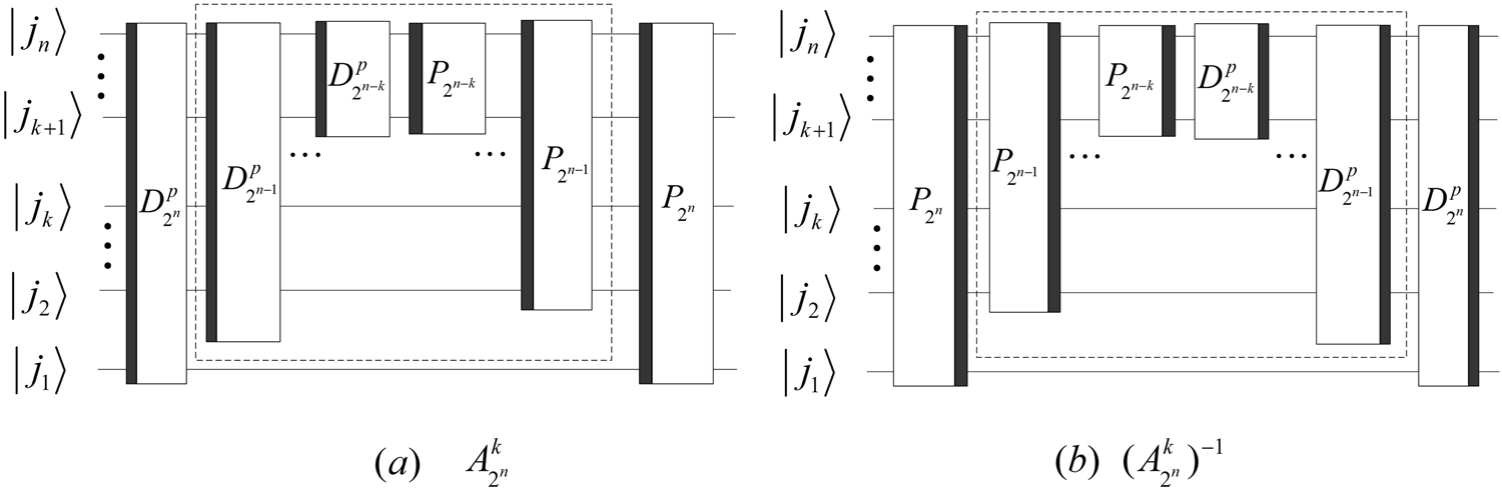
The quantum circuits of ${A}_{{2}^{n}}^{k}$ and ${({A}_{{2}^{n}}^{k})}^{-1}$. The dashed boxes in (**a**,**b**) implement ${A}_{{2}^{n-1}}^{k-1}$ and ${({A}_{{2}^{n-1}}^{k-1})}^{-1}$, respectively.

The implement circuits of the above DQWPT are shown in Fig. 20. Substituting the kernel matrix ${W}_{{2}^{n}}$ with ${T}_{{2}^{n}}$ in (35) and (36), we obtain that the (*k* + 1)-th iterations of the DQWPT and its inverse based on the periodization extension are

42 $$\{\begin{array}{rcl}{A}_{{2}^{n}}^{k} & = & {P}_{{2}^{n-1},2}({A}_{{2}^{n-1}}^{k-1}\otimes {I}_{2}){D}_{{2}^{n}}^{p},\\ {({A}_{{2}^{n}}^{k})}^{-1} & = & {Q}_{{2}^{n}}{({D}_{{2}^{n}})}^{-1}({({A}_{{2}^{n-1}}^{k-1})}^{-1}\otimes {I}_{2}){P}_{2,{2}^{n-1}}\end{array}$$

with the initial values ${A}_{{2}^{n-k}}^{0}={T}_{{2}^{n-k}}$, ${({A}_{{2}^{n-k}}^{0})}^{-1}={({T}_{{2}^{n-k}})}^{-1}$, 1 ≤ *k* < *n* − 1 and their implementation circuits shown in Fig. 21.

**Figure 22:**
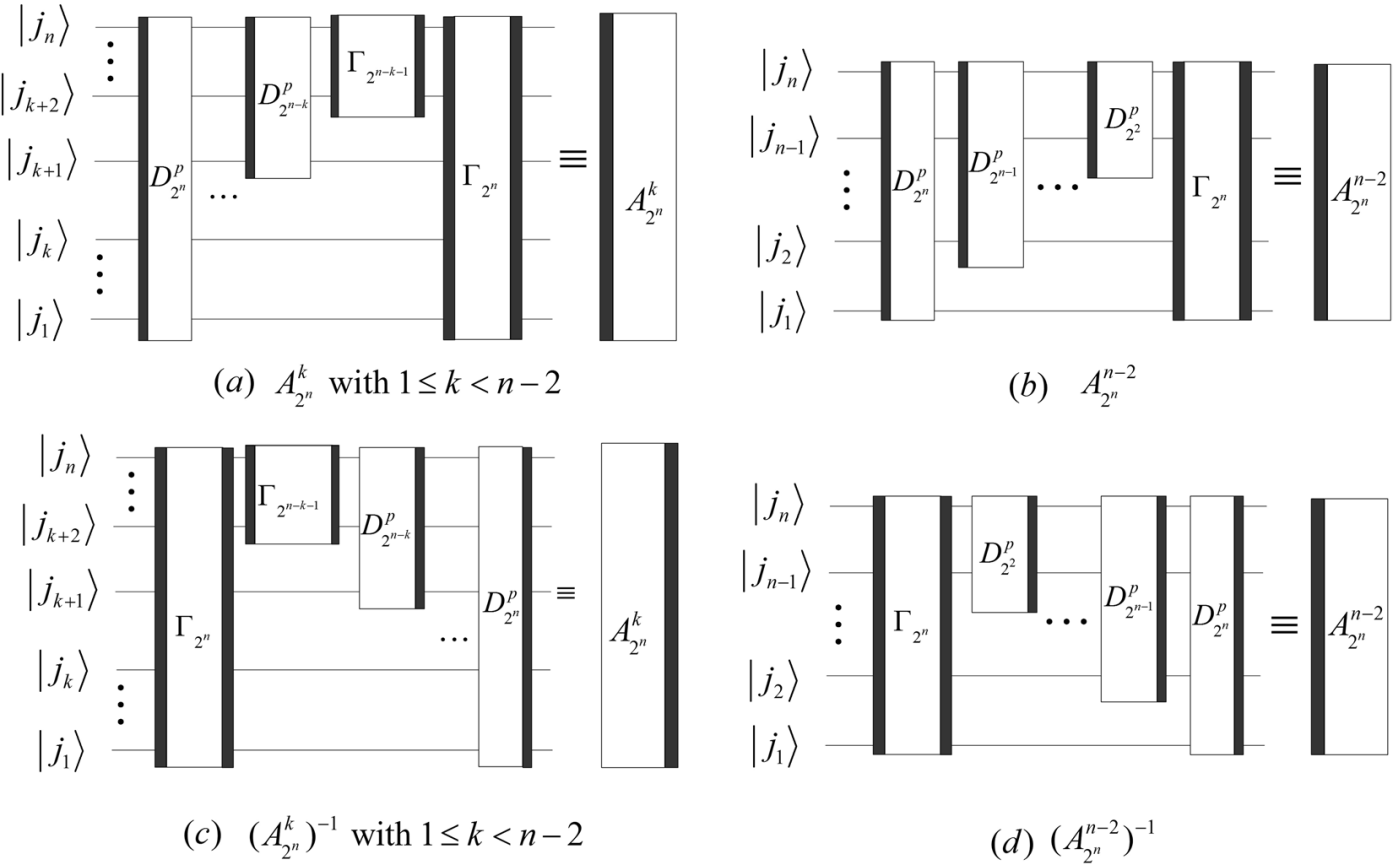
The simplified circuits of ${A}_{{2}^{n}}^{k}$ and ${({A}_{{2}^{n}}^{k})}^{-1}$ with 1 ≤ *k* < *n* − 1.

Using ${{\rm{\Gamma }}}_{{2}^{n}}$, the quantum circuit of ${A}_{{2}^{n}}^{k}$ and ${({A}_{{2}^{n}}^{k})}^{-1}$ can be simplified and shown in Fig. 22.

We analyze the complexity of the above DQWPT and suppose $r=\lfloor n/2\rfloor$.

From Figs 19 and 20, we calculate the complexity of ${T}_{{2}^{n}}$ by

43 $${C}^{p}({T}_{{2}^{n}})={C}^{p}({({T}_{{2}^{n}})}^{-1})={C}^{P}({Q}_{{2}^{n}})+{C}^{p}({D}_{{2}^{n}})+{C}^{P}({P}_{{2}^{n-1},2})=\sum _{i=1}^{n-1}\,2C({N}_{n}^{i})+10.$$

Applying lemma 1, corollary 1, corollary 2 and theorem 1, we obtain

44 $${C}^{p}({T}_{{2}^{n}})={C}^{p}({({T}_{{2}^{n}})}^{-1})=\{\begin{array}{l}52.5{n}^{2}-216n+38,\,{\rm{n}}\,{\rm{is}}\,{\rm{even}},\\ 52.5{n}^{2}-201n+16.5,\,{\rm{n}}\,{\rm{is}}\,{\rm{odd}}.\end{array}$$

We calculate the quantum cost of ${T}_{{2}^{n}}$ by

45 $$C({T}_{{2}^{n}})=C({({T}_{{2}^{n}})}^{-1})=C({Q}_{{2}^{n}})+C({D}_{{2}^{n}})+C({P}_{{2}^{n-1},2})={C}^{p}({T}_{{2}^{n}})+3n-9.$$

Let $${\varphi }_{2}(k)=\{\begin{array}{l}3,k=n-2\\ 6,1\le k < n-2,\end{array}$$ the time complexity of DQWPT is

46 $${C}^{p}({A}_{{2}^{n}}^{k})={C}^{p}({({A}_{{2}^{n}}^{k})}^{-1})=\sum _{i=1}^{n-k-1}\,2(k+1)C({N}_{n}^{i})+\sum _{i=n-k}^{n-1}\,2(n-i)C({N}_{n}^{i})+4(k+1)+{\varphi }_{2}(k).$$

For instance,

47 $${C}^{p}({A}_{{2}^{n}}^{n-2})={C}^{p}({({A}_{{2}^{n}}^{n-2})}^{-1})=\{\begin{array}{l}5{n}^{3}+21{n}^{2}-163.5n+63,\,{\rm{n}}\,{\rm{is}}\,{\rm{even}},\\ 5{n}^{3}+23.5{n}^{2}-168.5n+51,\,{\rm{n}}\,{\rm{is}}\,{\rm{odd}},\end{array}$$

and

48 $${C}^{p}({A}_{{2}^{n}}^{1})={C}^{p}({({A}_{{2}^{n}}^{1})}^{-1})=\{\begin{array}{l}70{n}^{2}-316n+115,\,{\rm{n}}\,{\rm{is}}\,{\rm{even}},\\ 70{n}^{2}-296n+85,\,{\rm{n}}\,{\rm{is}}\,{\rm{odd}}.\end{array}$$

The costs of ${A}_{{2}^{n}}^{k}$ and ${({A}_{{2}^{n}}^{k})}^{-1}$ are

49 $$C({A}_{{2}^{n}}^{k})=C({({A}_{{2}^{n}}^{k})}^{-1})={C}^{p}({A}_{{2}^{n}}^{k})-{\varphi }_{2}(k)+\lfloor \frac{n-k-1}{2}\rfloor +\lfloor \frac{n}{2}\rfloor .$$

## The 2D and 3D QWPTs

Firstly, we briefly describe NASS to represent 2D images and 3D videos. The NASS state $|{\psi }_{2}\rangle$ of an image can be represented by

50 $$|{\psi }_{2}\rangle =\sum _{{x}_{m}=0}^{{2}^{m}-1}\,\sum _{{y}_{k}=0}^{{2}^{k}-1}\,{\theta }_{{x}_{m},{y}_{k}}|{x}_{m}\rangle |{y}_{k}\rangle ,$$

where $|{x}_{m}\rangle =|{i}_{n}\ldots {i}_{k+1}\rangle$ and $|{y}_{k}\rangle =|{i}_{k}\ldots {i}_{1}\rangle$ are the X-axis and Y-axis of the image, ${\theta }_{{x}_{m},{y}_{k}}$ represents the color of the pixel in the coordinate $|{x}_{m}\rangle$ $|{y}_{k}\rangle$, and *n* = *m* + *k*.

The NASS state $|{\psi }_{3}\rangle$ of a video can represented by

51 $$|{\psi }_{3}\rangle =\sum _{{x}_{m}=0}^{{2}^{m}-1}\,\sum _{{y}_{k}=0}^{{2}^{k}-1}\,\sum _{{t}_{h}=0}^{{2}^{h}-1}\,{\theta }_{{x}_{m},{y}_{k},{t}_{h}}|{x}_{m}\rangle |{y}_{k}\rangle |{t}_{h}\rangle ,$$

where $|{x}_{m}\rangle =|{i}_{n}\ldots {i}_{h+k+1}\rangle$, $|{y}_{k}\rangle =|{i}_{h+k}\ldots {i}_{h+1}\rangle$ and $|{t}_{h}\rangle =|{i}_{h}\ldots {i}_{1}\rangle$ are the X-axis, Y-axis and time-axis of a video, and *n* = *m* + *k* + *h*.

**Figure 23:**
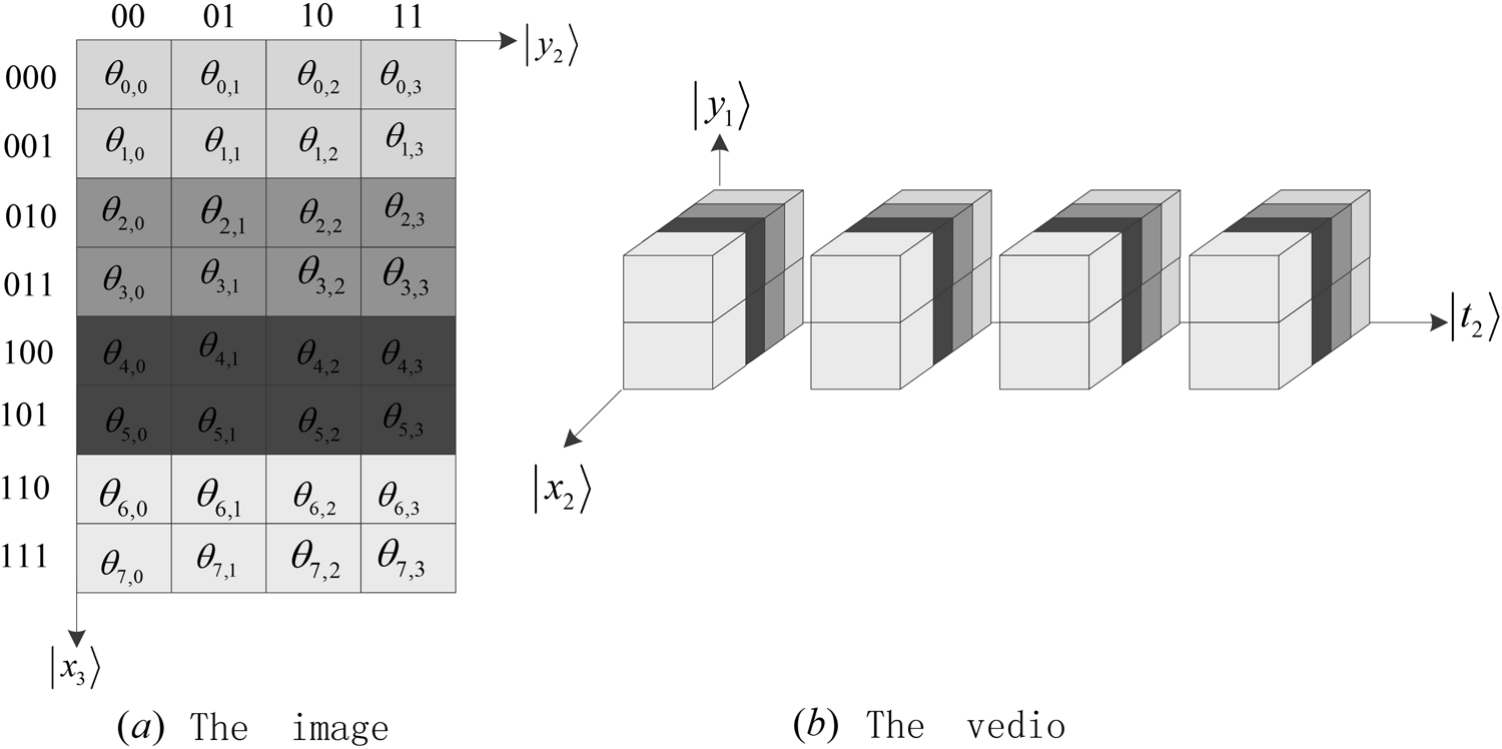
The image and the video.

More details are shown in our previous work^6^. For instance, the NASS state

52 $$|{\psi }_{2}\rangle =\sum _{{x}_{3}=0}^{{2}^{3}-1}\,\sum _{{y}_{2}=0}^{{2}^{2}-1}\,{\theta }_{{x}_{3},{y}_{2}}|{x}_{3}\rangle |{y}_{2}\rangle ={\theta }_{0,0}|000\rangle |00\rangle +\cdots +{\theta }_{7,3}|111\rangle |11\rangle $$

represents the color image of 8 × 4 (height multiplies weight) as shown in (a) of Fig. 23.

The NASS state

53 $$|{\psi }_{3}\rangle =\sum _{{x}_{2}=0}^{{2}^{2}-1}\,\sum _{{y}_{1}=0}^{1}\,\sum _{{t}_{2}=0}^{{2}^{2}-1}\,{\theta }_{{x}_{m},{y}_{k},{t}_{h}}|{x}_{2}\rangle |{y}_{1}\rangle |{t}_{2}\rangle ={\theta }_{0,0,0}|00\rangle |0\rangle |00\rangle +\cdots +{\theta }_{3,1,3}|11\rangle |1\rangle |11\rangle $$

represents the video with four frames as shown in (b) of Fig. 23, where each frame is a 4 × 2 image.

The same string can have different meanings corresponding to different data types in classic computers. For instance, a binary string 0100001 can represent a char ‘A’ or a number 65. Similarly, using the circuit in^6^, we can store an image (shown in (a) of Fig. 23) or a video (shown in (b) of Fig. 23) in the following state

54 $$|\psi \rangle =\sum _{i=0}^{{2}^{5}-1}\,{\theta }_{i}|i\rangle .$$

Meanwhile, the priori knowledge ‘*x*_3_, *y*_2_’ or ‘*x*_2_, *y*_1_, *t*_2_’ is equivalent to a data type, implying an image or a video stored in the state $|\psi \rangle$.

A natural image with size of 2^*n*^ × 2^*m*^ can be expressed as an angle matrix

55 $${{\rm{\Lambda }}}_{{2}^{n},{2}^{m}}=[\begin{array}{cccc}{\theta }_{0,0} & {\theta }_{0,1} & \cdots  & {\theta }_{0,{2}^{m}-1}\\ {\theta }_{1,0} & {\theta }_{1,1} & \cdots  & {\theta }_{1,{2}^{m}-1}\\ \vdots  & \vdots  & \cdots  & \vdots \\ {\theta }_{{2}^{n}-1,0} & {\theta }_{{2}^{n}-1,1} & \cdots  & {\theta }_{{2}^{n}-1,{2}^{m}-1}\end{array}],$$

where *θ*_*x*,*y*_ is the color information of the pixel on the coordinate (*x*, *y*) and an example is shown in Fig. 23.

Thus, the 2D wavelet transform on ${{\rm{\Lambda }}}_{{2}^{n}{\mathrm{,2}}^{m}}$ is defined as

56 $$wt2({{\rm{\Lambda }}}_{{2}^{n},{2}^{m}})={W}_{{2}^{n}}\times {{\rm{\Lambda }}}_{{2}^{n},{2}^{m}}\times {W}_{{2}^{m}}^{T},$$

where ${W}_{{2}^{n}}$ and ${W}_{{2}^{m}}$ are 2^*n*^ × 2^*n*^ and 2^*m*^ × 2^*m*^ wavelet transforms, respectively

An image can be stored in the state NASS $|{\psi }_{2}\rangle$ in (50) by using a quantum circuit in the literature^6^. Suppose that the function $f(\,\cdot \,)$ is equivalent to the quantum circuit implementing the storage of the image ${{\rm{\Lambda }}}_{{2}^{n}{\mathrm{,2}}^{m}}$, that is,

57 $$f({{\rm{\Lambda }}}_{{2}^{n},{2}^{m}})=|{\psi }_{2}\rangle =[\begin{array}{c}{B}_{0}^{T}\\ \vdots \\ {B}_{{2}^{n}-1}^{T}\end{array}],$$

where ${B}_{j}=[{\theta }_{j,0}\,{\theta }_{j,1}\,\cdots \,{\theta }_{j,{2}^{m}-1}]$ is the row vector of ${{\rm{\Lambda }}}_{{2}^{n}{\mathrm{,2}}^{m}}$ and 0 ≤ *j* ≤ 2^*n*^ − 1.

Applying the function $f(\,\cdot \,)$ on ${{\rm{\Lambda }}}_{{2}^{n}{\mathrm{,2}}^{m}}\times {W}_{{2}^{m}}^{T}$, the result is

58 $$f({{\rm{\Lambda }}}_{{2}^{n},{2}^{m}}\times {W}_{{2}^{m}}^{T})=({I}_{{2}^{n}}\otimes {W}_{{2}^{m}})f({{\rm{\Lambda }}}_{{2}^{n},{2}^{m}}).$$

Using the perfect shuffle permutation ${P}_{{2}^{m}{\mathrm{,2}}^{n}}$, we obtain

59 $$f({{\rm{\Lambda }}}_{{2}^{n},{2}^{m}})={P}_{{2}^{m},{2}^{n}}f({({{\rm{\Lambda }}}_{{2}^{n},{2}^{m}})}^{T}).$$

Then, we have

60 $$f({W}_{{2}^{n}}{{\rm{\Lambda }}}_{{2}^{n},{2}^{m}})=({W}_{{2}^{n}}\otimes {I}_{{2}^{m}})f({{\rm{\Lambda }}}_{{2}^{n},{2}^{m}}),$$

61 $$f({W}_{{2}^{n}}{{\rm{\Lambda }}}_{{2}^{n},{2}^{m}}{W}_{{2}^{m}}^{T})=({W}_{{2}^{n}}\otimes {W}_{{2}^{m}})f({{\rm{\Lambda }}}_{{2}^{n},{2}^{m}}).$$

Then, the 2D QWPT of ${{\rm{\Lambda }}}_{{2}^{n}{\mathrm{,2}}^{m}}$ is given by

62 $$f(wt2({{\rm{\Lambda }}}_{{2}^{n},{2}^{m}}))=({W}_{{2}^{n}}\otimes {W}_{{2}^{m}})|{\psi }_{2}\rangle .$$

A video of 2^*p*^ frames of size 2^*n*^ × 2^*m*^ corresponds to the following angle matrix.

63 $${A}_{{2}^{n},{2}^{m},{2}^{p}}=({{\rm{\Lambda }}}_{{2}^{n},{2}^{m}}^{1},{{\rm{\Lambda }}}_{{2}^{n},{2}^{m}}^{2},\cdots ,{{\rm{\Lambda }}}_{{2}^{n},{2}^{m}}^{{2}^{p}}),$$

where the angle matrix ${{\rm{\Lambda }}}_{{2}^{n}{\mathrm{,2}}^{m}}^{k}$ is the *k*-th frame.

We firstly define the following DWPTs: ${W}^{x}(\,\cdot \,)$, ${W}^{y}(\,\cdot \,)$ and ${W}^{t}(\,\cdot \,)$.

64 $$\{\begin{array}{l}{W}^{x}({A}_{{2}^{n},{2}^{m},{2}^{p}})=({W}_{{2}^{n}}{{\rm{\Lambda }}}_{{2}^{n},{2}^{m}}^{1},\,\cdots ,{W}_{{2}^{n}}{{\rm{\Lambda }}}_{{2}^{n},{2}^{m}}^{{2}^{p}}),\\ {W}^{y}({A}_{{2}^{n},{2}^{m},{2}^{p}})=({{\rm{\Lambda }}}_{{2}^{n},{2}^{m}}^{1}{W}_{{2}^{m}}^{T},\,\cdots ,{{\rm{\Lambda }}}_{{2}^{n},{2}^{m}}^{{2}^{p}}{W}_{{2}^{m}}^{T}),\\ {W}^{t}({A}_{{2}^{n},{2}^{m},{2}^{p}})=({C}_{{2}^{n},{2}^{m}}^{1},{C}_{{2}^{n},{2}^{m}}^{2},\,\cdots ,{C}_{{2}^{n},{2}^{m}}^{{2}^{p}}),\end{array}$$

with the row vectors

65 $$\{\begin{array}{l}[{C}_{x,y}^{1}\,{C}_{x,y}^{2}\,\cdots \,{C}_{x,y}^{{2}^{p}}]={u}_{x,y}\times {W}_{{2}^{m}}^{T},\\ {u}_{x,y}=[{\theta }_{x,y}^{1}\,{\theta }_{x,y}^{2}\,\cdots \,{\theta }_{x,y}^{{2}^{p}}],\end{array}$$

where ${C}_{x,y}^{j}$ and ${\theta }_{x,y}^{j}$ are the elements of the matrices ${C}_{{2}^{n}{\mathrm{,2}}^{m}}^{j}$ and ${{\rm{\Lambda }}}_{{2}^{n}{\mathrm{,2}}^{m}}^{j}$ on the position (*x*, *y*), respectively.

Next, the 3D DFPT of ${A}_{{2}^{n}{\mathrm{,2}}^{m}{\mathrm{,2}}^{p}}$ can be defined as

66 $$wt3({A}_{{2}^{n},{2}^{m},{2}^{p}})={W}^{t}({W}^{y}({W}^{x}({A}_{{2}^{n},{2}^{m},{2}^{p}}))).$$

Similarly, we utilize the equivalent function of the quantum circuit to create the NASS state of ${A}_{{2}^{n}{\mathrm{,2}}^{m}{\mathrm{,2}}^{p}}$

67 $$|{\psi }_{3}\rangle =f({A}_{{2}^{n},{2}^{m},{2}^{p}})={[{u}_{0,0}\cdots {u}_{0,{2}^{m}-1}{u}_{1,0}\cdots {u}_{1,{2}^{m}-1}\cdots {u}_{{2}^{n}-1,0}\cdots {u}_{{2}^{n}-1,{2}^{m}-1}]}^{T},$$

where the row vector *u*_*x*,*y*_ is shown in equation (65).

Applying the function $f(\,\cdot \,)$ on ${F}^{t}({A}_{{2}^{n}{\mathrm{,2}}^{m}{\mathrm{,2}}^{p}})$, ${F}^{y}({A}_{{2}^{n}{\mathrm{,2}}^{m}{\mathrm{,2}}^{p}})$ and ${F}^{x}({A}_{{2}^{n}{\mathrm{,2}}^{m}{\mathrm{,2}}^{p}})$ respectively, we have the following three equations.

68 $$\{\begin{array}{rcl}f({W}^{t}({A}_{{2}^{n},{2}^{m},{2}^{p}})) & = & ({I}_{{2}^{n+m}}\otimes {W}_{{2}^{p}})|{\psi }_{3}\rangle ,\\ f({W}^{y}({A}_{{2}^{n},{2}^{m},{2}^{p}})) & = & ({I}_{{2}^{n}}\otimes {W}_{{2}^{m}}\otimes {I}_{{2}^{p}})|{\psi }_{3}\rangle ,\\ f({W}^{x}({A}_{{2}^{n},{2}^{m},{2}^{p}})) & = & ({W}_{{2}^{n}}\otimes {I}_{{2}^{m}}\otimes {I}_{{2}^{p}})|{\psi }_{3}\rangle .\end{array}$$

Therefore, we derive the 3D QWPT of ${A}_{{2}^{n}{\mathrm{,2}}^{m}{\mathrm{,2}}^{p}}$

69 $$f(wt3({A}_{{2}^{n},{2}^{m},{2}^{p}}))=({W}_{{2}^{n}}\otimes {W}_{{2}^{m}}\otimes {W}_{{2}^{p}})|{\psi }_{3}\rangle .$$

**Figure 24:**
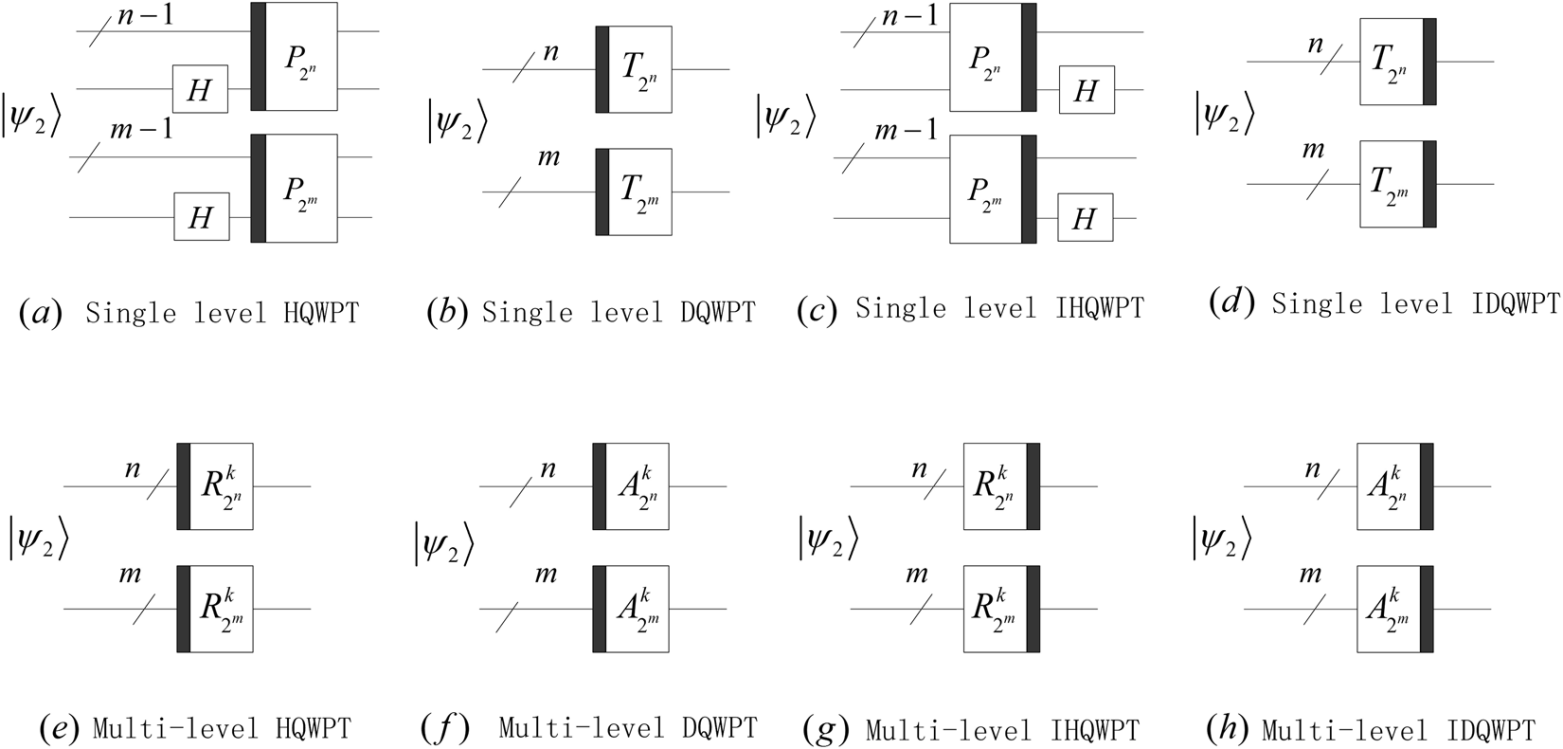
The quantum circuits of the 2D QWPT and IQWPT with 1 ≤ *k* ≤ min(*m*, *n*) − 1 in (**e**,**f**), 1 ≤ *k* ≤ min(*m*, *n*) − 2 in (**f**,**h**).

**Figure 25:**
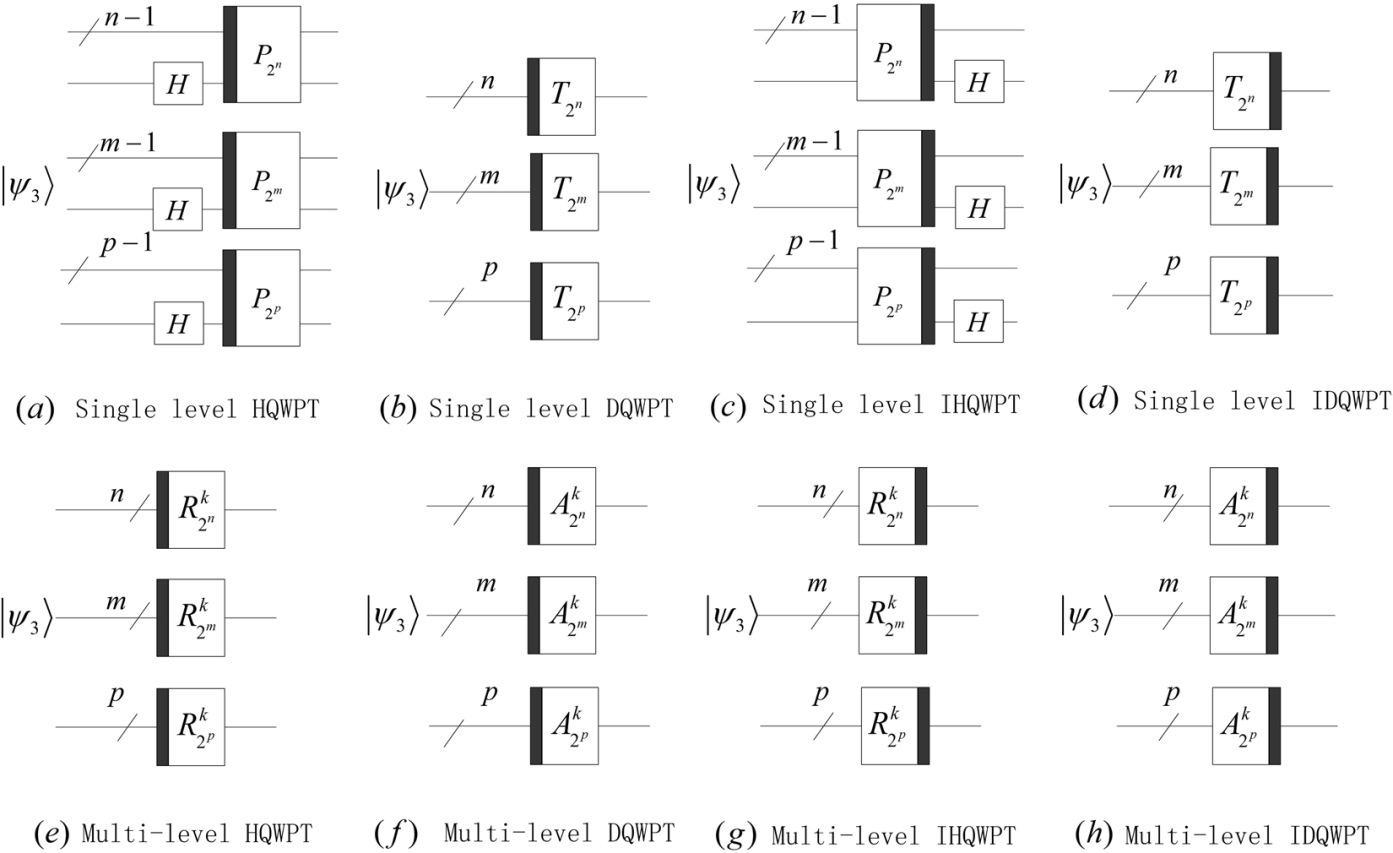
The quantum circuits of the 2D QWPT and IQWPT with 1 ≤ *k* ≤ min(*m*, *n*, *p*) − 1 in (**e**,**f**), 1 ≤ *k* ≤ min(*m*, *n*, *p*) − 2 in (**f**,**h**).

Substituting our proposed 1D QWPT into equations (62) and (69), we obtain 2D HQWPT, 2D DQWPT, 3D HQWPT and 3D DQWPT. Furthermore, their circuits can be designed in Figs 24 and 25.

## Simulation Experiments

In the absence of a quantum computer to implement our proposed QWPTs, experiments of quantum signals are simulated on a classical computer. The quantum signals are stored in quantum states (i.e., column vectors) and the QWPTs are implemented using unitary matrices in Matlab (the R2010bversion).

### Simulation experiments of the 1D HQWPT and DQWPT

Consider a quantum state

70 $$|v\rangle =\frac{1}{\sqrt{\sum _{i=0}^{2047}{({v}_{i})}^{2}}}\,{[{v}_{0}{v}_{1}\cdots {v}_{2047}]}^{T}$$

as an input signal of the QWT, where *v*_*k*_ = *d*(*k*/2048), *k* = 0, …, 2047, and $d(t)=\sqrt{t(1-t)}\,\sin \,(\frac{2\pi \,\ast \,1.05}{t+0.05})$.

For simply, we can take a vector

71 $$S={[{v}_{0}{v}_{1}\cdots {v}_{2047}]}^{T}$$

as the input signal of simulation experiments, which is according with the state $|v\rangle$ without the normalized item.

For convenience, let the single-level HQWPT and DQWPT be

72 $${R}_{{2}^{11}}^{0}={P}_{{2}^{n-1},2}({I}_{{2}^{n-1}}\otimes H),{A}_{{2}^{11}}^{0}={T}_{{2}^{11}}.$$

**Figure 26:**
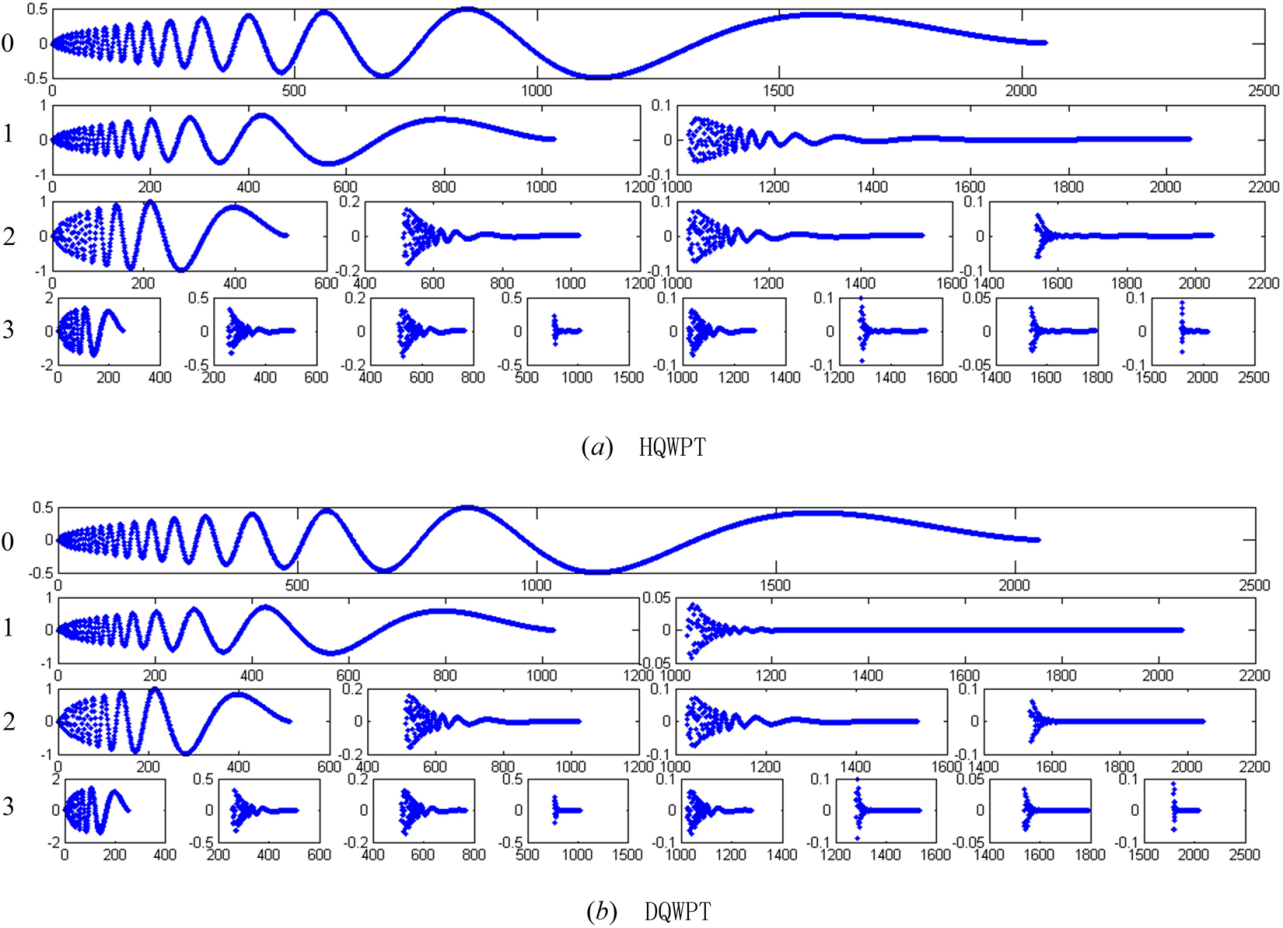
The simulation results of the first 3 levels of HQWPT and DQWPT. The left number *i* refers to *i*-level QWT with 1 ≤ *i* ≤ 10, and *i* = 0 refers to the input signal.

Applying multi-level HQWPT ${R}_{{2}^{11}}^{k},k=0,1,\ldots 10$ and multi-level DQWPT ${A}_{{2}^{11}}^{k},k=0,1,\ldots 9$ to the input signal *S* in Eq. (71), the simulation results of the first 3 levels are shown in Fig. 26 with multi-windows. Table 1 shows the comparison of simulation experiments of our proposed QWPT and the function of the WPT in Matlab using the 2-norm function $norm()$. The symbols in this table are listed as follows:

73 $$\{\begin{array}{l}{M}_{1}^{k}={R}_{{2}^{11}}^{k}\times S,0\le k\le 10,\\ {M}_{2}^{k}={({R}_{{2}^{11}}^{k})}^{-1}\times {M}_{1}^{k},0\le k\le 10,\\ {M}_{3}^{k}={A}_{{2}^{11}}^{k}\times S,0\le k\le 9,\\ {M}_{4}^{k}={({A}_{{2}^{11}}^{k})}^{-1}\times {M}_{3}^{k},0\le k\le 9.\end{array}$$

**Table 1:** The simulation results of the HQWT, IHQWT and HWT.

level	${\boldsymbol{norm}}\,({{\boldsymbol{M}}}_{{\bf{1}}}^{{\boldsymbol{k}}}{\boldsymbol{-}}{{\boldsymbol{M}}}_{{\bf{5}}}^{{\boldsymbol{k}}}){\boldsymbol{\times }}1{{\bf{0}}}^{{\bf{13}}}$	${\boldsymbol{norm}}\,({\boldsymbol{S}}{\boldsymbol{-}}{{\boldsymbol{M}}}_{{\bf{2}}}^{{\boldsymbol{k}}}){\boldsymbol{\times }}1{{\bf{0}}}^{{\bf{13}}}$	${\boldsymbol{norm}}\,({{\boldsymbol{M}}}_{{\bf{3}}}^{{\boldsymbol{k}}}{\boldsymbol{-}}{{\boldsymbol{M}}}_{{\bf{6}}}^{{\boldsymbol{k}}}){\boldsymbol{\times }}1{{\bf{0}}}^{{\bf{10}}}$	${\boldsymbol{norm}}\,({\boldsymbol{S}}{\boldsymbol{-}}{{\boldsymbol{M}}}_{{\bf{4}}}^{{\boldsymbol{k}}}){\boldsymbol{\times }}1{{\bf{0}}}^{{\bf{13}}}$
1	0	0.0211	0.0057	0.0257
2	0.0228	0.0583	0.0128	0.0511
3	0.0252	0.0643	0.0230	0.0798
4	0.0361	0.0619	0.0374	0.1006
5	0.0311	0.0716	0.0574	0.1371
6	0.0693	0.0736	0.0842	0.1662
7	0.0505	0.1118	0.1168	0.2402
8	0.0960	0.1518	0.1564	0.2575
9	0.1325	0.1874	0.1963	0.3040
10	0.1982	0.2899	0.1560	0.3623
11	0.2497	0.3981	—	—

The function $wpdec(S,k+1,^{\prime} db1^{\prime} )$ in Matlab performs a (*k* + 1)-level HWPT to return a wavelet packet tree. Next, we get the coefficients of the nodes of the wavelet packet tree using the function $wpcoef()$ to construct a vector ${M}_{5}^{k}$. similarly, we obtain a a vector ${M}_{6}^{k}$ of a (*k* + 1)-level DWPT based on the periodization extension by the $wpdec(S,k+1,^{\prime} db2^{\prime} )$ and $wpcoef()$.

### Simulation experiments of the 2D HQWPT and DQWPT

**Figure 27:**
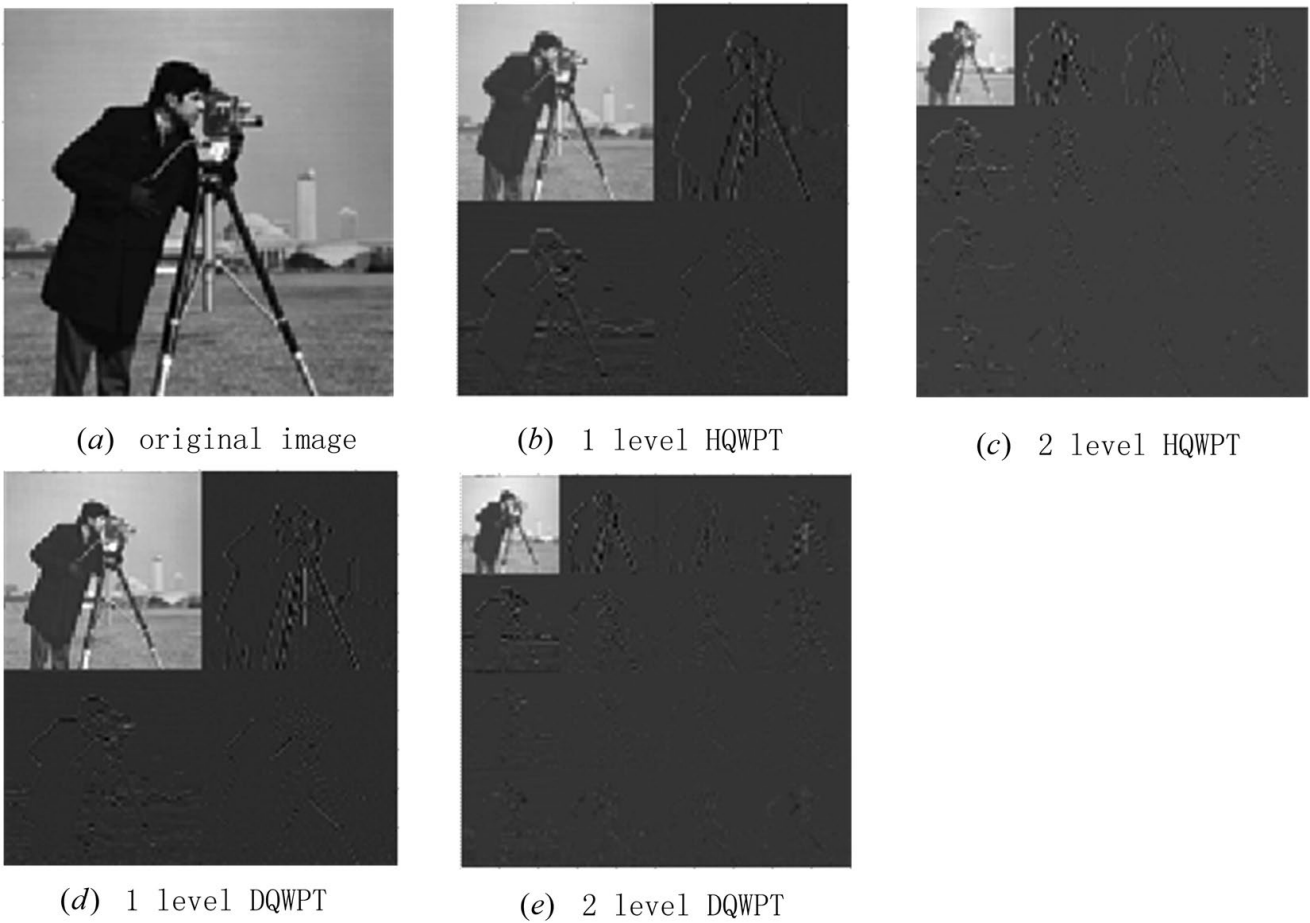
The simulation results of the first 2 levels of HQWPT and DQWPT.

An angle matrix Λ_*g*_ is given by

74 $${{\rm{\Lambda }}}_{g}=\frac{\pi {C}_{g}}{2\times {2}^{8}-2}{A}_{g},$$

where *A*_*g*_ is the 128 × 128 matrix of the gray-scale image shown in Fig. 27(a), and *C*_*g*_ is a constant corresponding to the image.

The NASS state $|{\psi }_{2}\rangle =f({{\rm{\Lambda }}}_{g})$ can be regarded as a column vector, where the function $f(\,\cdot \,)$ is defined in equation (57). Applying the *k* + 1 level 2D HQWPT and DQWPT on the image *A*_*g*_, respectively, the results are

75 $$\{\begin{array}{l}{Q}_{1}^{k}=\frac{2\times {2}^{8}-2}{\pi {C}_{g}}{f}^{-1}(({R}_{{2}^{7}}^{k}\otimes {R}_{{2}^{7}}^{k})|{\psi }_{2}\rangle ),\\ {Q}_{3}^{k}=\frac{2\times {2}^{8}-2}{\pi {C}_{g}}{f}^{-1}(({A}_{{2}^{7}}^{k}\otimes {A}_{{2}^{7}}^{k})|{\psi }_{2}\rangle ),\end{array}$$

where ${f}^{-1}(\,\cdot \,)$ is the inverse function of $f(\,\cdot \,)$, which converts a column vector into a 2-dimension matrix.

The simulation results are shown in Fig. 27 and Table 2. The rest symbols in Table 2 are: ${Q}_{2}^{k}=|{\psi }_{2}\rangle -$ $({({R}_{{2}^{7}}^{k})}^{-1}\otimes {({R}_{{2}^{7}}^{k})}^{-1})[({R}_{{2}^{7}}^{k}\otimes {R}_{{2}^{7}}^{k})|{\psi }_{2}\rangle ]$, ${Q}_{2}^{k}=|{\psi }_{2}\rangle -({({A}_{{2}^{7}}^{k})}^{-1}\otimes {({A}_{{2}^{7}}^{k})}^{-1})\,[({A}_{{2}^{7}}^{k}\otimes {A}_{{2}^{7}}^{k})|{\psi }_{2}\rangle ]$. Similarly with the 1D HQWPT and DQWPT, matrices ${Q}_{5}^{k}$ and ${Q}_{6}^{k}$ are created using the functions $wpdec2({A}_{g},k+1,^{\prime} db1^{\prime} )$, $wpdec2({A}_{g},k+1,^{\prime} db2^{\prime} )$ and $wpcoef()$, respectively.

**Table 2:** The simulation results of the 2D HQWT, IHQWT and HWT.

level	${\boldsymbol{norm}}\,({{\boldsymbol{Q}}}_{{\bf{1}}}^{{\boldsymbol{k}}}{\boldsymbol{-}}{{\boldsymbol{Q}}}_{{\bf{5}}}^{{\boldsymbol{k}}}){\boldsymbol{\times }}1{{\bf{0}}}^{{\bf{9}}}$	${\boldsymbol{norm}}\,(|{{\boldsymbol{\psi }}}_{{\bf{2}}}\rangle {\boldsymbol{-}}{{\boldsymbol{Q}}}_{{\bf{2}}}^{{\boldsymbol{k}}}){\boldsymbol{\times }}1{{\bf{0}}}^{{\bf{13}}}$	${\boldsymbol{norm}}\,({{\boldsymbol{Q}}}_{{\bf{3}}}^{{\boldsymbol{k}}}{\boldsymbol{-}}{{\boldsymbol{Q}}}_{{\bf{6}}}^{{\boldsymbol{k}}}){\boldsymbol{\times }}1{{\bf{0}}}^{{\bf{8}}}$	${\boldsymbol{norm}}\,(|{{\boldsymbol{\psi }}}_{{\bf{2}}}\rangle {\boldsymbol{-}}{{\boldsymbol{Q}}}_{{\bf{4}}}^{{\boldsymbol{k}}}){\boldsymbol{\times }}1{{\bf{0}}}^{{\bf{14}}}$
1	0.0011	0.0041	0.0669	0.0427
2	0.0022	0.0093	0.0128	0.0880
3	0.0026	0.0104	0.2641	0.1837
4	0.0099	0.0125	0.3917	0.2904
5	0.0218	0.0242	0.5851	0.6436
6	0.0679	0.0518	0.9105	0.8180
7	0.1218	0.1079	—	—

### Simulation experiments of the 3D HQWPT and DQWPT

**Figure 28:**
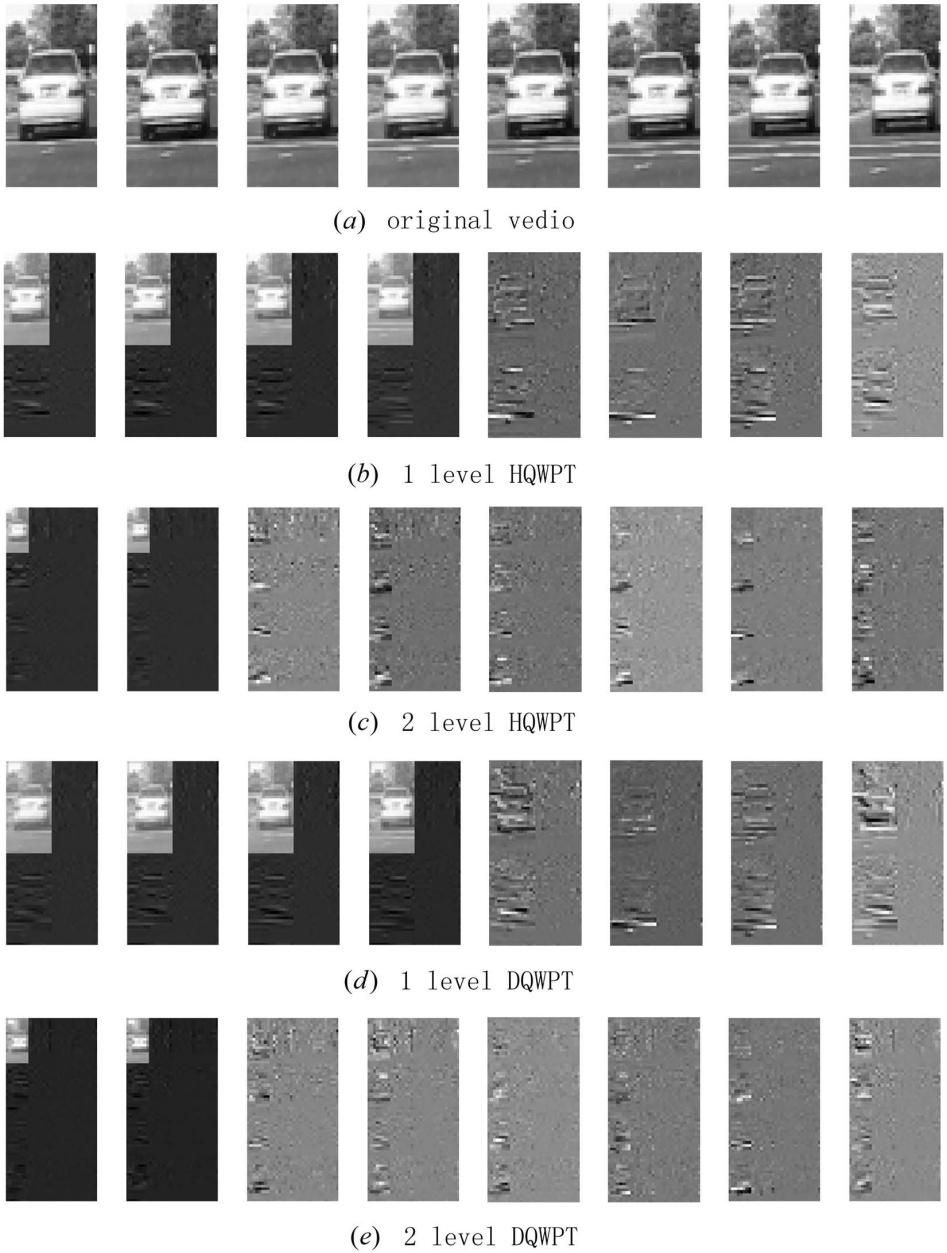
The simulation results of the first 2 levels of the 3D HQWPT and DQWPT.

An angle matrix Λ_*c*_ is given by

76 $${{\rm{\Lambda }}}_{c}=\frac{\pi {C}_{c}}{2\times {2}^{8}-2}{V}_{t},$$

where *V*_*t*_ is the 64 × 64 × 4 matrix of the video shown in (a) of Fig. 28, and *C*_*c*_ is a constant corresponding to the video.

The NASS state $|{\psi }_{3}\rangle =f({{\rm{\Lambda }}}_{c})$ can be regarded as a column vector, where the function $f(\,\cdot \,)$ is defined in equation (67). Applying the *k* + 1 level 3D HQWPT and DQWPT on the video *V*_*t*_, respectively, the results are

77 $$\{\begin{array}{l}{V}_{1}^{k}=\frac{2\times {2}^{8}-2}{\pi {C}_{g}}{f}^{-1}(({R}_{{2}^{6}}^{k}\otimes {R}_{{2}^{6}}^{k}\otimes {R}_{{2}^{6}}^{k})|{\psi }_{3}\rangle ),\\ {V}_{3}^{k}=\frac{2\times {2}^{8}-2}{\pi {C}_{g}}{f}^{-1}(({A}_{{2}^{6}}^{k}\otimes {A}_{{2}^{6}}^{k}\otimes {A}_{{2}^{6}}^{k})|{\psi }_{3}\rangle ),\end{array}$$

where ${f}^{-1}(\,\cdot \,)$ converts a column vector into a 3-dimension matrix.

The simulation results are shown in Fig. 28 and Table 3. Since there are no functions of the 3D WPT, we realize *wt*3 in (66) using the functions $wpdec\mathrm{2()}$ and $wpdec()$ and note ${V}_{5}^{k}$ and ${V}_{6}^{k}$ as results of 3D HWPT and DWPT, respectively. The rest symbols in Table 2 are ${V}_{2}^{k}=|{\psi }_{3}\rangle -({({R}_{{2}^{6}}^{k})}^{-1}\otimes {({R}_{{2}^{5}}^{k})}^{-1}\otimes {({R}_{{2}^{3}}^{k})}^{-1})\,[({R}_{{2}^{6}}^{k}\otimes {R}_{{2}^{5}}^{k}\otimes {R}_{{2}^{3}}^{k})\,|{\psi }_{3}\rangle ]$ and ${V}_{4}^{k}=|{\psi }_{3}\rangle -({({A}_{{2}^{6}}^{k})}^{-1}\otimes {({A}_{{2}^{5}}^{k})}^{-1}\otimes {({A}_{{2}^{3}}^{k})}^{-1})\,[({A}_{{2}^{6}}^{k}\otimes {A}_{{2}^{5}}^{k}\otimes {A}_{{2}^{3}}^{k})\,|{\psi }_{3}\rangle ]$.

**Table 3:** The simulation results of the 3D HQWT, IHQWT and HWT.

level	${\boldsymbol{norm}}\,({{\boldsymbol{V}}}_{{\bf{1}}}^{{\boldsymbol{k}}}{\boldsymbol{-}}{{\boldsymbol{V}}}_{{\bf{5}}}^{{\boldsymbol{k}}}){\boldsymbol{\times }}1{{\bf{0}}}^{{\bf{10}}}$	${\boldsymbol{norm}}\,(|{{\boldsymbol{\psi }}}_{3}\rangle {\boldsymbol{-}}{{\boldsymbol{V}}}_{{\bf{2}}}^{{\boldsymbol{k}}}){\boldsymbol{\times }}1{{\bf{0}}}^{{\bf{14}}}$	${\boldsymbol{norm}}\,({{\boldsymbol{V}}}_{{\bf{3}}}^{{\boldsymbol{k}}}{\boldsymbol{-}}{{\boldsymbol{V}}}_{{\bf{6}}}^{{\boldsymbol{k}}}){\boldsymbol{\times }}1{{\bf{0}}}^{{\bf{8}}}$	${\boldsymbol{norm}}\,(|{{\boldsymbol{\psi }}}_{3}\rangle {\boldsymbol{-}}{{\boldsymbol{V}}}_{{\bf{4}}}^{{\boldsymbol{k}}}){\boldsymbol{\times }}1{{\bf{0}}}^{{\bf{14}}}$
1	0.0164	0.0488	0.1787	0.0714
2	0.0702	0.1449	0.3328	0.2005
3	0.2171	0.2044	—	—

Analyzing the above simulation experiments, we conclude that our proposed HQWPT, IHQWPT, DQWPT and IDQWPT can implement decompositions and reconstructions of the Haar wavelet and D4 wavelet, respectively. The simulation results of our proposed HQWPT and DQWPT, which are equal to the corresponding WPTs without consideration of truncation error on machine computing, show our proposed QWPTs are correct.

## Conclusion and Future Works

This article has constructed the iteration equations of multi-level and multi-dimensional QWPTs by GTP and PSP. The iteration equations include HQWPT, DQWPT based on the periodization extension and their inverse transforms for the first time, which ensure the theoretical correctness of our proposed QWPTs. Next, we have designed circuits of the proposed QWPTs. The precise analysis of the quantum costs and the time complexities of circuits prove that our proposed QWPTs are of high-efficiency. For instance, the time complexities of the multi-level HQWPT and DQWPT at most are 6 and (5*n*^3^ + *O*(*n*^2^)) on 2^*n*^, respectively. In contrast, the classical fast WPTs need *O*(*n*2^*n*^) basic operations to implement the discrete wavelet transform^21,32^. Thus, our proposed QWPT can exponentially speed up the computation of the wavelet transform in comparison to the one on a classical computer. The simulation results show that our proposed QWTs are correct and effective. In summary, the proposed QWPTS and IQWPTs can implement effective decompositions and reconstructions of 1D signals, 2D images and 3D vedio, respectively. Therefore, the article provide a feasible scheme for the WPT to be applied in QIP.

Studies of quantum wavelet packet are still in their infancy. Multi-level and multi-dimension wavelet transforms play an important role in classical image and signal processing, therefore, their quantum versions will be significant and core tool algorithms for quantum image and signal processing. Our future works are how to use these wavelet transforms to implement some complex operations, such as quantum image and signal compression, and quantum image and signal denoising.
